# Light-Responsive Molecular Release from Cubosomes Using Swell-Squeeze Lattice Control

**DOI:** 10.1021/jacs.2c08583

**Published:** 2022-10-12

**Authors:** Beatrice E. Jones, Elaine A. Kelly, Nathan Cowieson, Giorgio Divitini, Rachel C. Evans

**Affiliations:** † Department of Materials Science and Metallurgy, University of Cambridge, 27 Charles Babbage Road, Cambridge CB3 0FS, United Kingdom; ‡ Diamond Light Source, Harwell Science and Innovation Campus, Didcot, Oxfordshire OX11 0QX, United Kingdom

## Abstract

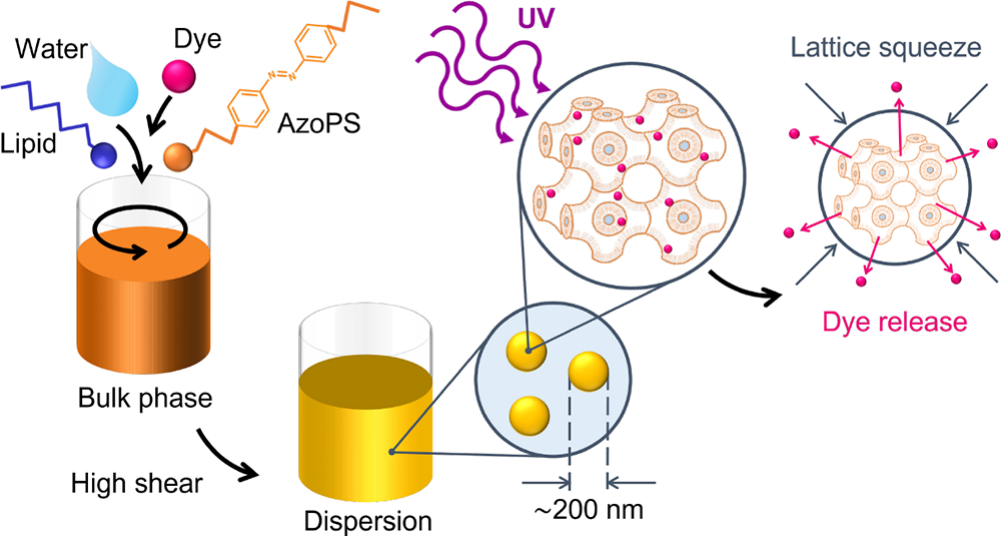

Stimuli-responsive materials are crucial to advance controlled delivery systems for drugs and catalysts. Lyotropic liquid crystals (LLCs) have well-defined internal structures suitable to entrap small molecules and can be broken up into low-viscosity dispersions, aiding their application as delivery systems. In this work, we demonstrate the first example of light-responsive cubic LLC dispersions, or cubosomes, using photoswitchable amphiphiles to enable external control over the LLC structure and subsequent on-demand release of entrapped guest molecules. Azobenzene photosurfactants (AzoPS), containing a neutral tetraethylene glycol head group and azobenzene-alkyl tail, are combined (from 10–30 wt %) into monoolein-water systems to create LLC phases. Homogenization of the bulk LLC forms dispersions of particles, ∼200 nm in diameter with internal bicontinuous primitive cubic phases, as seen using small-angle X-ray scattering and cryo-transmission electron microscopy. Notably, increasing the AzoPS concentration leads to swelling of the cubic lattice, offering a method to tune the internal nanoscale structure. Upon UV irradiation, AzoPS within the cubosomes isomerizes within seconds, which in turn leads to squeezing of the cubic lattice and a decrease in the lattice parameter. This squeeze mechanism was successfully harnessed to enable phototriggerable release of trapped Nile Red guest molecules from the cubosome structure in minutes. The ability to control the internal structure of LLC dispersions using light, and the dramatic effect this has on the retention of entrapped molecules, suggests that these systems may have huge potential for the next-generation of nanodelivery.

## Introduction

Emerging methods to control the delivery of small molecules have wide-spread applications spanning from pharmaceuticals, notably for COVID-19 mRNA vaccines, to catalytic reactions, agriculture, and food. Targeting the release of payloads ensures that they are used in a direct manner, which can reduce waste and unwanted side-effects.^1^ To enable this, materials that can entrap molecular payloads and release them on-demand using an external stimulus are required as delivery systems. One method for molecular entrapment is to use lyotropic liquid crystals (LLCs), which are formed from the self-assembly of amphiphiles on the addition of a solvent and possess long-range orientational order.^2^ These ordered networks have complex, nanoscale internal structures, which restrict outward diffusion of guest molecules.^3^ Furthermore, the amphiphilic nature of LLCs mean that a variety of molecules of differing hydrophilicities can be contained within them, including drugs,^4^ catalysts,^5^ or medical imaging agents.^6^ However, bulk LLC mesophases are often viscous, making them challenging to administer. To aid their use, they can be broken up in excess aqueous solution to form low-viscosity dispersions of nanoparticles, while retaining the internal order necessary for controlled delivery.^6,7^

Monoolein (MO) is an amphiphilic lipid commonly used to create host LLCs due to its propensity to form stable dispersions, as well as its biocompatibility and biodegradability.^3^ It can form a variety of LLC phases depending on the solvent concentration and polarity of molecular additives.^8,9^ These phases can be broken up to form colloidal dispersions of particles typically between 200 and 300 nm in diameter,^6^ most commonly using a high-shear input (sonication or homogenization),^10^ with additional interfacial stabilization.^11^ The retention and release of entrapped guest molecules are governed by the internal structure of the dispersed LLC particles.^12^ Liposomes, which are vesicles with an outer lipid bilayer shell, have been extensively researched and clinically implemented for controlled delivery applications.^13^ However, the simple structure can lead to problems with premature drug leakage and fast release rates.^14^ To combat this, hexagonal (hexosomes) and cubic (cubosomes) LLC phases are of particular interest, as the complex two- and three-dimensional interfaces between the water channels and the amphiphile bilayer slow the diffusion of entrapped species.^15,16^ The release of guest molecules can be controlled by the LLC dimensions, which directly affect diffusion rates.^4^ Furthermore, the larger lipid surface area in comparison to simple liposomes allows a higher guest payload to be incorporated.^14^

However, undirected release of active molecules results in wasted payload away from the target site, which can even manifest as harm in the case of toxic drugs.^17^ As such, methods to control the time and position of release using an external stimulus are needed. Light is particularly attractive as a stimulus as its intensity, wavelength, duration, and spatial position can be easily adjusted. Light-responsive LLC dispersions have previously been created through addition of metallic nanoparticles, which induce photothermal phase changes;^18,19^ however, the toxicity of nanoparticle additions remains a concern.^20^ Alternatively, LLCs can be built from amphiphiles, which contain a photoswitchable group.^21−28^ Of these, azobenzene photosurfactants (AzoPS) have been the most extensively studied.^29^ Despite concerns over its potential toxicity, a promising recent work has shown the capability to produce biocompatible azobenzene derivatives.^30^ On irradiation with UV light, azobenzene photoisomerizes from the *trans* (*E*) to the *cis* (*Z*) state, forming a photostationary state (PSS) of mostly *cis*-isomers, with a composition dependent on irradiation wavelength, solvent, temperature, and chemical structure.^31^ Reverse isomerization can be triggered using visible light, giving a second PSS of mostly *trans*-isomers, or fully, using heat.^32,33^ Isomerization leads to a change in shape^34^ and polarity^35^ of the molecule, which, when combined into amphiphiles, modifies the molecular geometry and hydrophilicity.^29^ This has been shown to have a knock-on effect on the interfacial- and self-assembly of AzoPS at low concentrations, both with charged, ionic,^36^ and neutral head groups.^37^ As such, AzoPS have been investigated for applications such as: DNA compaction,^38^ microfluidics,^39^ foam stability,^40^ and micellar entrapment and release.^41,42^

At higher concentrations, there have been several reports of the self-assembly of AzoPS into higher-order bulk LLC phases using both charged^24^ and neutral surfactants;^23,27,28,43,44^ however, research has focused on the latter, due to a greater number of hydrogen bonding sites, thought to aid LLC formation.^45^ In work by Peng *et al.*, neutral AzoPS molecules, with an oligooxyethylene head and azobenzene-alkyl tail, formed both lamellar and hexagonal LLCs with photoisomerization resulting in a loss of the hexagonal phase.^23,26^ Houston *et al.* further demonstrated the ability to control LLC phase formation, including lamellar and hexagonal, using the structure, concentration, and temperature in systems of neutral AzoPS, all of which showed loss of LLC order on isomerization.^25^

Some controlled release mechanisms using light-responsive AzoPS have been reported. Aleandri *et al.* created light-responsive bulk hexagonal and cubic LLC mesophases by introducing small amounts of an Azo-MO analogue into LLC phases containing a mixture of MO and oleic acid.^46^ The authors observed that photoisomerization increased the diffusivity of the hexagonal LLC, leading to an increased rate of release of an entrapped hydrophilic dye. However, interestingly, small-angle X-ray scattering (SAXS) results showed no structural difference in the LLCs upon photoisomerization, suggesting that the Azo-MO analogue did not impart significant organizational change at the concentrations studied (<5 wt %). Looking to nanoparticle dispersions, Pernpeintner *et al.* formed simple liposomes using phosphatidylcholines that were modified with an azobenzene group in the tail.^47^ For clinical applications, the low tissue penetration of UV light remains an issue. To combat this, the group further functionalized the azo-phospholipid using tetra-ortho-chloro substitution to red-shift the isomerization wavelength from UV (365 nm) to red (660 nm).^48^ Using this approach, light-driven drug release was achieved both *in vitro* and *in vivo*, showing the potential for azobenzene-stimulated release in human tissue. However, to the best of our knowledge, there have been no previous reports of AzoPS-induced light-responsivity in nanoparticles exhibiting internal LLC order, whose increased dimensional control could set them apart as the next-generation of nanodelivery devices.

**Figure 1:**
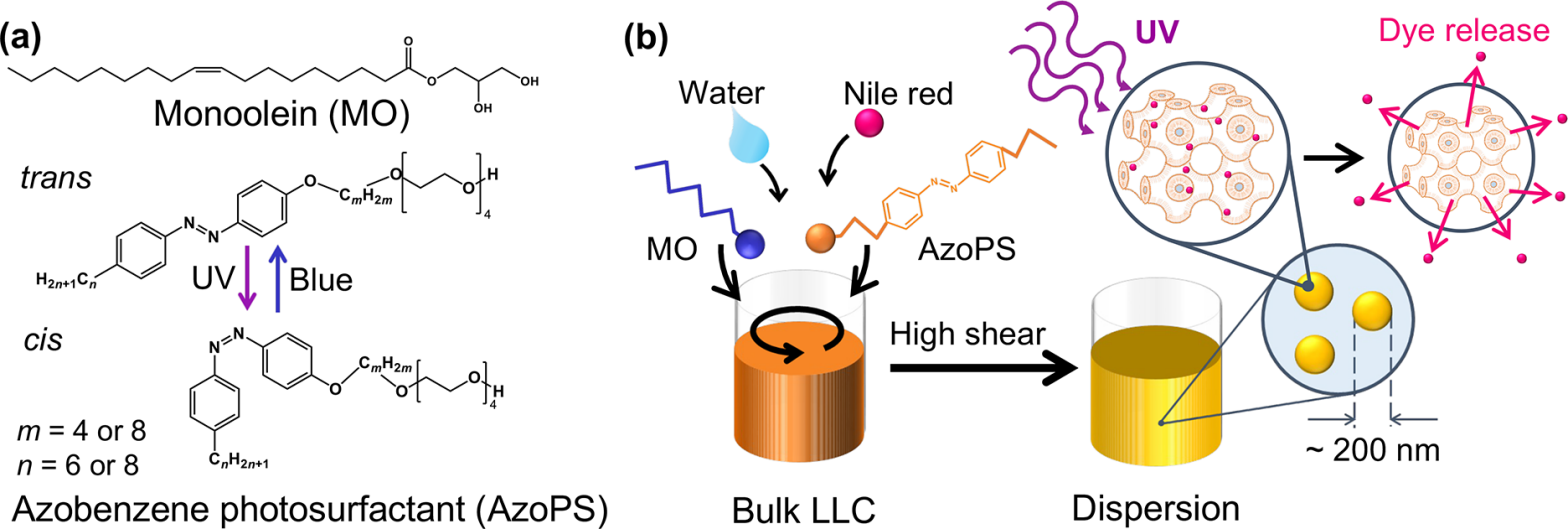
Creation of light-responsive LLC nanoparticles. (a) Chemical structures of MO and AzoPS molecules used, showing isomerization with UV (365 nm) and blue (455 nm) light. Alkyl chain lengths *m* and *n* were varied to give two different structures: C_6_AzoC_4_E_4_ and C_8_AzoC_8_E_4_. (b) Schematic diagram showing the formation of the bulk LLC, dispersed nanoparticles, and effects of isomerization: decreasing the cubic lattice parameter and triggering release of the dye, Nile Red.

Herein, we demonstrate for the first time light-responsive cubosomes, which show measurable change in the internal structure on photoisomerization. Bulk LLCs were prepared from a mixture of MO, water, and a neutral AzoPS dopant, with known LLC-forming abilities^25^ (Figure 1a), which transform into cubosomes under application of a shear force (Figure 1b). Using SAXS, polarized optical microscopy (POM) and cryo-transmission electron microscopy (TEM), we show that the internal structure of the cubosomes can be swelled by modifying the bulk LLC precursor composition (i.e., AzoPS or water wt %). Moreover, photoisomerization squeezes the cubic lattice, leading to triggerable release of entrapped guest species (e.g., Nile Red) at rapid timescales in comparison to diffusion rates, enhancing their application as stimuli-responsive delivery materials.

## Results and Discussion

### Creating Bulk LLCs Containing Light-Responsive AzoPS

We first investigated the formation of LLCs within bulk MO-AzoPS-water mixtures by SAXS. Two different AzoPS structures were investigated, both containing a tetraethylene glycol head group but with differing alkyl spacer (*m* = 4 or 8) and alkyl tail (*n* = 6 or 8) lengths, subsequently referred to as C_6_AzoC_4_E_4_ and C_8_AzoC_8_E_4_ (Figure 1a). AzoPS were loaded at 20 wt % with respect to MO, and the water content was 20 wt % with respect to total amphiphile mass. The resulting concentration of AzoPS was above the critical micelle concentration for both structures (Supporting Information, Table S1). A reference sample of MO-water (20 wt %) was also prepared.

**Figure 2:**
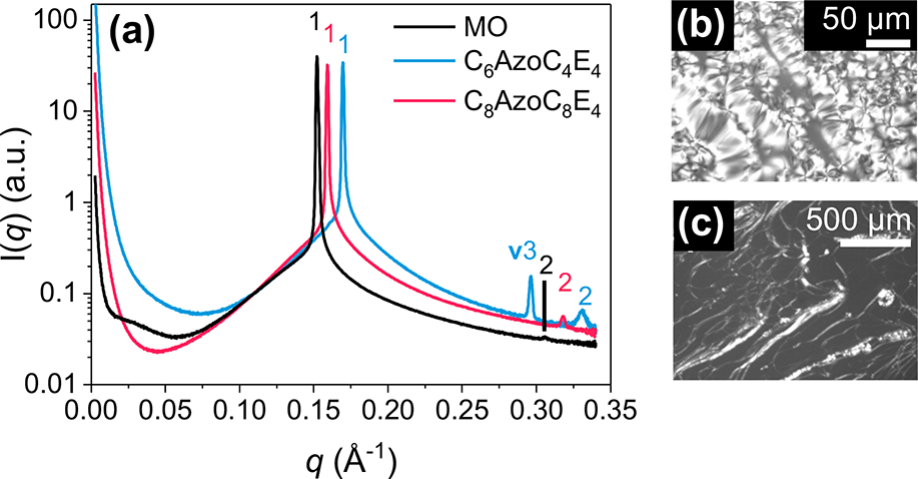
Bulk LLC phase formation in MO-AzoPS-water mixtures at 25 °C. The AzoPS is in the native, *trans* form. (a) SAXS plots for bulk LLCs containing: MO, either C_6_AzoC_4_E_4_ or C_8_AzoC_8_E_4_ (20 wt % with respect to MO) or no AzoPS (labeled MO), and water (20 wt % with respect to total amphiphile mass). Peak ratios show the formation of hexagonal and lamellar LLCs for samples containing C_6_AzoC_4_E_4_ and C_8_AzoC_8_E_4_, respectively. POM images confirm the assignment of hexagonal and lamellar phases from the (b) fan (C_6_AzoC_4_E_4_) and (c) stripy patterns (C_8_AzoC_8_E_4_), respectively.

MO-water shows sharp Bragg diffraction peaks in the ratio of 1:2 (Figure 2a), indicating the formation of a lamellar LLC phase, as expected at this composition and temperature (25 °C).^49^ This phase assignment is supported by a characteristic streaky pattern in the POM images (Supporting Information, Figure S1b). On incorporation of AzoPS, there is a shift in the diffraction peaks in the SAXS patterns (Figure 2a), which depends on the tail length of the AzoPS molecule. A characteristic peak ratio of 1:√3:2 suggests the formation of a hexagonal phase in the ternary MO-C_6_AzoC_4_E_4_-water system.^4^ A birefringent fan pattern under POM supports this phase assignment (Figure 2b). In contrast, the analogous C_8_AzoC_8_E_4_ system exhibits SAXS peaks in the ratio of 1:2, indicating the formation of a lamellar LLC mesophase, visible as a striped pattern under POM (Figure 2c).

The sensitivity of the LLC phase to the AzoPS structure demonstrates that there is an interaction between the MO and AzoPS in the LLC, and that they are not forming separate self-assembled structures. The preference for different LLC phases by the two AzoPS structures can be rationalized using critical packing parameter (CPP) considerations for the spontaneous curvature of the amphiphile films.^50−52^ The CPP is defined as CPP = *v*/*a*_0_*l*_c,_ where *v* is the volume of the hydrophobic tail, *a*_0_ is the hydrophilic head group area, and *l*_c_ is the length of the hydrophobic chain.^53^ Due to the high amphiphile concentration with respect to water (80 wt %), inverse LLC phases, with negative curvature, are expected to form.^54^ Compared to MO, the AzoPS have a much larger head group areas, due to the four ethylene glycol groups, and also longer tail lengths. This results in smaller packing parameters (Supporting Information, Table S3), favoring lower-curvature phases when combined with MO, as modeled with similar neutral (but non-light responsive) surfactants.^55^ On decreasing the alkyl chain length from C_8_AzoC_8_E_4_ to C_6_AzoC_4_E_4_, there is a transition from the zero-curvature lamellar phase to the inverse hexagonal phase, with a higher negative curvature. This shows that the AzoPS chain length dominates the packing and spontaneous curvature of the amphiphile bilayer. This is consistent with previous findings for these AzoPS, where the ratio of alkyl chain length/head group area determined LLC formation;^25^ since the head group in both AzoPS here is the same, it is expected that the alkyl chain length determines the phase formation.

It is interesting to compare these results to LLC formation in AzoPS-water systems, without MO, from a previous work by Houston *et al*.^25^ C_6_AzoC_4_E_4_ forms a lamellar phase at 20 wt % water and 25 °C, showing that incorporation with MO, which has a higher CPP, increases the negative curvature of the amphiphile film, resulting in the inverse hexagonal phase. In comparison, C_8_AzoC_8_E_4_ remains as insoluble crystals on addition of 20 wt % water, at 25 °C. Addition to the MO matrix allows solubilization of the hydrophobic AzoPS to form the lamellar phase. Compared to the MO-water system, the incorporation of C_8_AzoC_8_E_4_ decreases the lamellar spacing (from 4.1 to 4.0 nm), visible as a shift in the SAXS peaks to higher *q* values. As the C_8_AzoC_8_E_4_ chain length is just under double that of MO (3.4 *cf.* 1.8 nm), this suggests that, in the lamellar phase, one AzoPS molecule spans across the MO bilayer. The result of this would be a decrease in the average lamellar spacing on incorporation of the AzoPS into the bilayer.

### Structural Characteristics of LLC Dispersions

The ability to break up bulk MO-AzoPS LLC phases to form low-viscosity dispersions under shear was next investigated. The AzoPS tail length, concentration of AzoPS (10–30 wt %, with respect to MO) and initial water concentration (10–40 wt % with respect to total amphiphile mass in the parent, bulk LLC phase) were all varied to probe changes in the LLC particles with composition. The bulks were then homogenized in a solution of Pluronic F-127 (0.3 wt %) to aid interfacial stability.

After homogenization, reference dispersions of MO-water formed particles with a mean Z-average hydrodynamic diameter, *D*_H_, between 130 and 190 nm (Table S4, Supporting Information), measured using dynamic light scattering (DLS). A small increase in particle size was observed on the incorporation of AzoPS into the LLCs, giving *D*_H_ typically between 159 and 220 nm. No clear trend was observed in the particle size with variation of the AzoPS tail length, concentration, or initial water concentration. An outlier of 468 ± 7 nm was measured for the dispersion containing 30 wt % C_8_AzoC_8_E_4_, indicating lower stability and agglomeration of particles with high AzoPS concentrations. This is accompanied by a general increase in the polydispersity index on increasing AzoPS concentration for both tail lengths (Table S4). Despite this, almost all dispersions had a particle polydispersity index below 0.3, which can be considered monodisperse for applications as lipid-based nanoparticles.^56^

The colloidal stability of the dispersions was determined by remeasuring the particle size after storage for 10 months at room temperature in the dark. None of the dispersions had visibly phase-separated during this period, with only a little agglomeration at the side of the vials (Supporting Information, Figure S4). However, for most samples, there was a significant decrease in the nanoparticle size, with *D*_H_ for most AzoPS-containing samples lying between 80 and 117 nm. A similar effect was observed for the reference MO-water dispersions, with *D*_H_ decreasing to 95–119 nm (SI, Table S5). This decrease in size was accompanied by an increase in the polydispersity index and can be attributed to dehydration of the particles over this time frame. Despite these variations in the particle size, it can be concluded that Pluronic F-127 provides sufficient stabilization to prevent large agglomerates over the timescale of months, giving long shelf-life potential in these systems.

**Figure 3:**
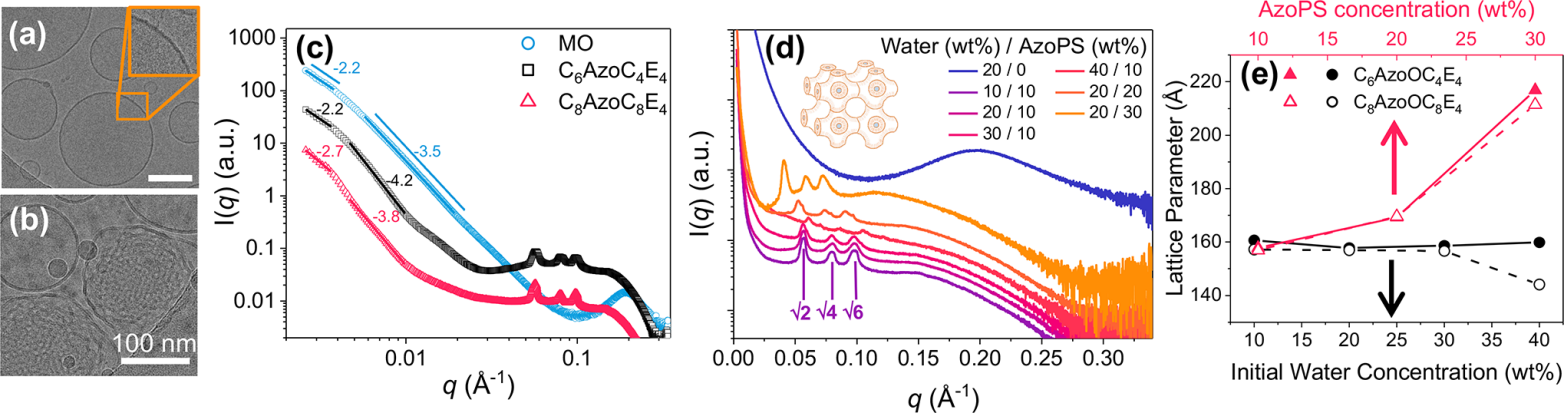
Internal and surface structure of MO-AzoPS particles in the *trans* state. TEM micrographs show (a) a double-ring vesicle surface in dispersions of MO-water (20 wt %), as magnified in the insert (orange box) and (b) evidence of internal order in MO-C_6_AzoC_4_E_4_ (10 wt %)-water (20 wt %) particles. (c) SAXS plots of log*I* vs log*q* for dispersions containing MO-water (10 wt %) and no AzoPS, C_6_AzoC_4_E_4_ (10 wt %) or C_8_AzoC_8_E_4_ (10 wt %) at 25 °C show two straight-line regions due to surface scattering. Curves have been offset for clarity. (d) SAXS curves for dispersions of MO with varying concentration of initial water and C_8_AzoC_8_E_4_ (*T* = 25 °C) show the formation of Bragg peaks in the ratio √2:√4:√6, characteristic of the inverse bicontinuous primitive cubic (*Im3m*) phase, shown in the schematic insert. Error bars were removed and curves were offset for clarity. (e) Variation of the cubic lattice parameter with concentration of initial water (at constant AzoPS concentration, 10 wt %) and C_6_AzoC_4_E_4_ or C_8_AzoC_8_E_4_ (20 wt % initial water). Note that errors from peak fitting are negligible within the scale of the lattice parameter.

Cryo-transmission electron microscopy was also used to image the particles. Dispersions of MO-water (20 wt %) showed a double-ring outer surface, attributable to the outer amphiphile bilayer of unilamellar vesicles (Figure 3a), as expected from the lamellar LLC in the bulk phase. In these vesicles, there was no sign of internal order. In contrast, MO-AzoPS-water dispersions exhibit visible internal structure in the micrographs (C_6_AzoC_4_E_4_ in Figure 3b and C_8_AzoC_8_E_4_ in Figure S5, SI). Surface scattering from these particles was further investigated by SAXS. Porod plots (log*I* vs log*q*) show two distinct straight-line regions, indicating scattering from two different length scales (Figure 3c).^57^ In the lowest *q* region, the scattering is proportional to ∼*q*^–2^, indicative of scattering from 2D sheets, attributable to the outer bilayer.^58^ Guinier analysis in this region was used to estimate the overall particle size, with resulting diameters (100–167 nm) in agreement those observed using DLS and TEM (SI, Table S6). At higher *q*, the scattering comes from the interface between the particles and the aqueous phase, with gradients of −3 and −4 corresponding to scattering from rough and smooth 3D fractal interfaces, respectively.^59^ This interface gradient decreases for the MO-AzoPS-water systems, indicating that a smoother fractal surface forms in AzoPS-containing particles compared to the MO-water reference. The gradient increase is accompanied by a shoulder region in the MO-AzoPS-water systems between *q* = 0.01 and 0.02 Å^–1^. This corresponds to features of length scales of 30–60 nm, which are visible as small vesicles in the cryo-TEM micrographs (Figure 3b), showing that there is some heterogeneity in the size and order of the dispersed particles.

Vesicles formed from MO-water dispersions showed no Bragg diffraction peaks in the SAXS patterns; only a broad peak at *q* = 0.2 Å^–1^ is observed (Figure 3d), corresponding to the real-space distance expected from the bilayer packing of MO molecules in the outer layer of the vesicles.^60^ Variation of the initial water content in the bulk phase (10–40 wt %) resulted in the same broad peak, showing that this had no effect on internal ordering (SI, Figure S6). However, upon incorporation of AzoPS into the MO-water dispersions, Bragg peaks with a *q* ratio of √2: √4:√6 become clearly apparent in the SAXS patterns (Figure 3d). This ratio is characteristic of the inverse bicontinuous primitive cubic (*Im3m*) LLC phase,^4^ corresponding to peaks of Miller indices of (110), (200), and (111), and indicates that cubosomes are present in the MO-AzoPS-water dispersions. The lattice parameter varied between 145 and 217 Å, which is comparable to other MO-based primitive cubosomes in the literature.^61^ This LLC phase was stable across the composition range tested (10–40 wt % initial water, 10–30 wt % AzoPS), for both chain lengths of AzoPS (for C_6_AzoC_4_E_4_ see SI, Figure S7) and across the temperature range of 25–55 °C (SI, Table S7). To the best of our knowledge, this is the first example where a light-responsive chemical moiety has been incorporated into dispersed nanoparticles to form a cubic LLC phase, which is highly desirable for future controlled release applications.

The bicontinuous primitive cubic LLC phase is associated with a high packing stress, where some amphiphiles are extended (around the water channels) and others are compressed (around lipid junctions). It has been observed previously that the inclusion of long-chain additives to MO can lower the packing stress by preferentially segregating to regions where the amphiphiles are in extension.^62^ In this case, the primitive cubic phase has been stabilized by the AzoPS addition, due to their longer chain length. Notably, variation in the lattice parameter was observed across the AzoPS composition range explored for both amphiphiles. The cubic lattice parameter (*a*) was calculated from *a* = 2π√(*h*^2^ + *k*^2^ + *l*^2^)/*q*_0_, where *q*_0_ is the peak center for the first observed Bragg peak and *h, k*, and *l* are the associated Miller indices (110).^61^ Increasing the concentration of AzoPS within the LLCs resulted in a stark increase in *a* (Figure 3e). In the *trans* state, both AzoPS have a longer hydrophobic tail and larger head-group area than MO and therefore a lower CPP. This favors a lower spontaneous curvature in the amphiphile bilayer and leads to swelling in the lattice at increasing concentrations.^63^ The increase in lattice size is further amplified by the increased thickness of the amphiphile bilayers from the longer chains.^64^

In comparison, on increasing the initial water concentration from 10 to 30 wt %, there was little variation in the lattice parameter (Figure 3e), which is directly related to the internal water content in the cubic phase through geometric packing analysis (SI, Table S8). This is as expected, as the internal water within the cubic lattice is free to move into the external solution. However, a decrease in lattice parameter, and therefore internal water content, was measured at the highest water concentration (40 wt %) with C_8_AzoC_8_E_4_. In the MO-water system, transition to a two-phase cubic + water region is expected at 40 wt % water.^49^ On incorporation of the more hydrophobic C_8_AzoC_8_E_4_, this could lead to the dispersion being formed in the two-phase region, which, in turn, could lead to preferential segregation of the C_8_AzoC_8_E_4_ into the aqueous phase. This would result in AzoPS depletion in the cubic lattice and a decrease in lattice parameter, as expected from the discussion above. This is also accompanied by a decrease in the intensity of the Bragg peaks, indicating a higher proportion of vesicles without internal cubic phases forming in the dispersion at this composition, providing an upper limit on the amount of water that can be incorporated before disordering occurs.

To retain LLC order, particularly at high concentrations, the choice of AzoPS structure was found to be crucial. On increasing the concentration of C_6_AzoC_4_E_4_ above 10 wt %, there was a significant decrease in the intensity of the Bragg peaks in the SAXS patterns (SI, Figure S7), indicating a disordering of the cubic LLC. This low order-retention was further demonstrated by repeating SAXS experiments for three samples made to the same composition (30 wt % C_6_AzoC_4_E_4_ and 20 wt % initial water), where no Bragg diffraction peaks were visible for two of the three samples (SI, Figure S9). In contrast, the disordering effect is less prevalent for dispersions containing the longer AzoPS, C_8_AzoC_8_E_4_, with repeat compositions (30 wt % C_8_AzoC_8_E_4_, 20 wt % initial water) retaining the inverse bicontinuous primitive cubic LLC phase to give reproducible results (SI, Figure S9). For both AzoPS structures, it was found that the peak intensity also increases on increasing the temperature to 55 °C. This was accompanied by a peak shift to higher *q* values, indicating a decrease in cubic lattice parameter (SI, Figure S10), as expected from the literature where higher temperatures lead to a dehydration of the surfactant head groups and an increase in the CPP.^4^ In the case of these MO-AzoPS systems, this acts to both contract the cubic lattice and increase the cubic phase stability at elevated temperatures. This implies that the AzoPS geometry, quantified by the CPP, is crucial in determining the stability of the cubic LLC phase. The estimated CPP at room temperature for C_8_AzoC_8_E_4_ is greater than C_6_AzoC_4_E_4_ (0.42 *cf.* 0.40, see SI, Table S3), implying that amphiphile geometries closer to that for MO (1.16)^65^ results in greater stability of the cubic phase and more reproducible cubosome formation. The LLC disordering at increased AzoPS concentrations provides an upper limit to the amount of light-sensitive material that can be added to these systems while retaining the internal order required for controlled molecular entrapment and release. The ability to tailor the stability through control of the light-responsive surfactant geometry is thus an important result for the subsequent optimization of these systems for molecular delivery applications.

### Isomerization within Light-Responsive LLC Dispersions

**Figure 4:**
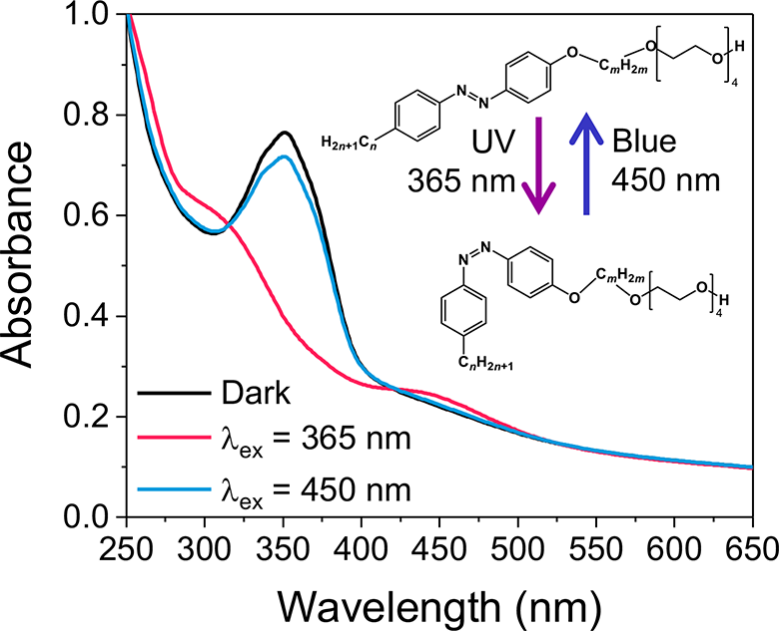
UV–vis absorption spectra show light-response within MO-AzoPS-water dispersions. A sample of MO-C_8_AzoC_8_E_4_ (20 wt %)-water (10 wt %) was diluted in water to prevent saturation of the detector (69 μM). The background scattering comes from the dispersed particles. Spectra on storage in the dark, irradiation with UV light (5 min) and subsequent blue light irradiation (5 min) indicate photoisomerization of the AzoPS within the dispersions from the *trans* to *cis* states.

Having formed LLC dispersions using AzoPS in their native, *trans* state, the effect of photoisomerization was next investigated. UV–vis absorption spectra for AzoPS within MO dispersions in the *trans* state show a peak at ∼350 nm, characteristic of the π–π* transition in azobenzene^31^ (Figure 4). We note that the spectra show a relatively high background due to Rayleigh scattering from the dispersed particles. When compared to the spectra for AzoPS diluted in water, introduction into LLC dispersions caused a red-shift in the absorption band by 19 nm (Figure S11), attributed to the formation of J-aggregates in the LLC structure.^66^ This formation of aggregates also contributes to the asymmetry in the π–π* peak (Figure 4), due to overlap of peak contributions from both aggregates and monomers.^67^

On irradiation with UV light (5 min), the *trans* peak decreases and two new peaks arise at λ_max_ = 319 and 450 nm (Figure 4, for C_6_AzoC_4_E_4_ see SI, Figure S12), attributed to the π–π* and previously forbidden *n*–π* transitions in the *cis* isomer, indicating that photoisomerization has occurred. The *trans* isomer can be partially recovered (94%) through irradiation with blue light. A photostationary state, containing a mixture of both isomers that dynamically switch between the two forms, is created on irradiation.^23^ The time needed to obtain a *cis*-dominant PSS and subsequent reversion to the *trans* state was determined using first-order kinetics, as in previous studies (see SI, Figures S13 and S14).^25^ Forward conversion occurred on the order of seconds for both AzoPS types (Table S9, SI). Reversion from the *cis* back to the *trans* state required longer irradiation times (∼10 s *cf.* ∼1 s for *trans*-*cis*), due to a combination of the lower irradiance from the blue LED and a lower absorption coefficient for the *n*–π* transition (for C_8_AzoC_8_E_4_, ε_*cis*,455nm_ = 340 m^2^mol^–1^*cf.* ε_*trans*,365nm_ = 1000 m^2^mol^–1^). Thermal reversion lifetimes, on storage in the dark, were on the order of hours (SI Table S9, Figures S15 and S16). On storage in the dark over the course of 1 month, the π–π* peak recovered at the same wavelength (∼350 nm). This indicates that there was minimal leaching of the AzoPS into solution over the course of the irradiation and relaxation cycle, which would result in a blue-shift of the peak as observed for the AzoPS alone in solution. With a view to controlled release applications, the high stability of the *cis* isomer over the course of hours is important for the storage and delivery of LLC particles to the target site. Combined with the rapid photoisomerization of AzoPS, this demonstrates a high degree of temporal control within these systems using light as an external stimulus.

### Effects of Isomerization on Structure and Size of MO-AzoPS Dispersions

**Figure 5:**
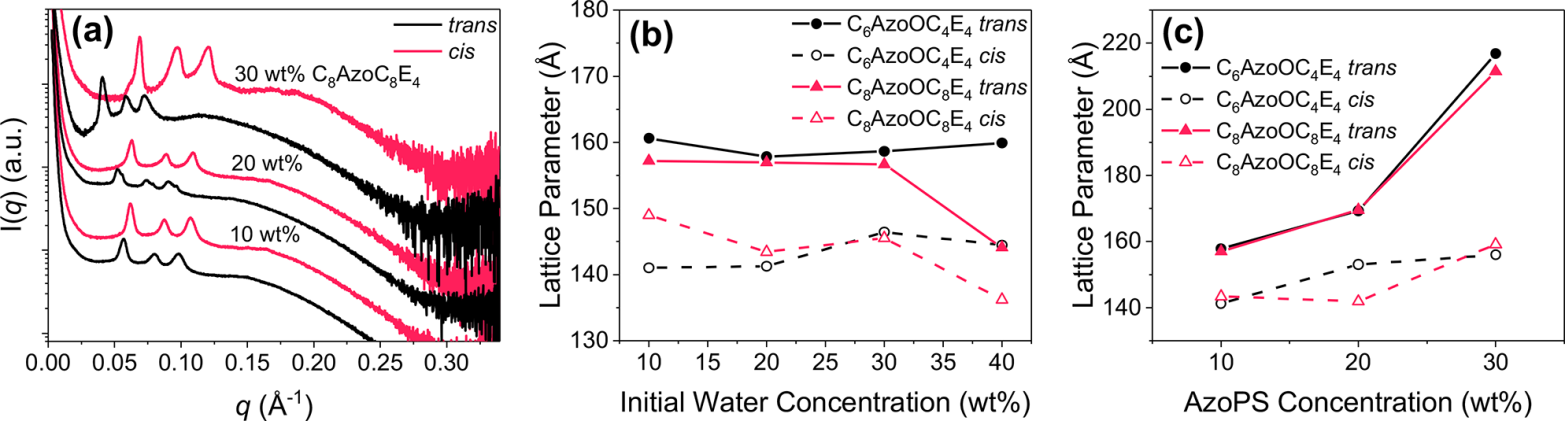
Decrease in the cubic lattice parameter on isomerization of AzoPS within LLC dispersions. (a) SAXS patterns for dispersions of MO-water (20 wt %) and varying concentrations of C_8_AzoC_8_E_4_ in the native, *trans* and irradiated, *cis* states. Error bars were removed and curves were offset for clarity. Plots show the variation in lattice parameter decrease on isomerization with varying concentrations of (b) AzoPS (20 wt % initial water) and (c) initial water (10 wt % AzoPS). Error bars from peak fitting are negligible within the scale of the lattice parameter graphs.

The effects of photoswitching on the size and structure of the nanoparticles present in MO-AzoPS-water dispersions was next investigated. Following irradiation to form the *cis*-dominant PSS, the SAXS patterns exhibited Bragg peaks of the same ratio, √2:√4:√6, across the whole composition range, showing the retention of the inverse bicontinuous primitive cubic phase (Figure 5 and SI, Figures S17–S19). However, the lattice parameter decreased across all samples on isomerization (Figure 5b,c). The measured change in the lattice parameter remained approximately stable on increasing the initial water concentration; however, a dramatic increase in the disparity between the *trans* and *cis*-state lattice parameters was observed at higher AzoPS concentrations (>20 wt %). A maximum decrease in lattice parameter of 39% was measured for the dispersion of MO with 30 wt % C_8_AzoOC_8_E_4_ and 20 wt % water. Repeat experiments for different samples of the same composition showed that this decrease in cubic lattice parameter was reproducible (see SI, Figure S20). We note that for an analogous sample containing C_6_AzoC_4_E_4_, low-intensity Bragg peaks were observed in the *trans* state, only showing peaks large enough to be assigned in one sample of three. Despite this, the little ordered material present in this sample showed high sensitivity to the isomeric state of the AzoPS, with a 28% decrease in the lattice parameter. Interestingly, in one sample, Bragg peaks only emerged after isomerization (see SI, Figure S20), further demonstrating that the cubic phase stability is dependent on AzoPS geometry.

On photoisomerization, “bending” of the AzoPS tail leads to an increase in tail volume and therefore CPP, increasing the spontaneous curvature of the amphiphile bilayer. This would result in contraction of the cubic lattice (and decrease in corresponding lattice parameter) as previously observed for structural changes in nonlight-responsive MO systems.^63^ Increasing the concentration of AzoPS within the LLC acts to magnify this effect, resulting in a larger change. The increased stability of the cubic LLC on isomerization can be attributed to the increase in the CPP, which is consistent with observations at increased temperatures and chain lengths in the *trans* samples. Despite this increase in cubic phase stability on isomerization, the greater disorder at high AzoPS concentrations, especially in C_6_AzoC_4_E_4_, will have a knock-on effect for the ability of these systems to entrap guest molecules. It is therefore crucial to strike a balance between achieving the maximum structural change, obtained from high concentrations of AzoPS, and retaining ordered LLC packing, which is sensitive to the disparity in geometries between MO and AzoPS. In this regard, the longer chain C_8_AzoOC_8_E_4_ showed a greater ability to retain LLC order at high concentrations, due to its higher CPP, maximizing the light-sensitivity and reproducibility of these systems.

**Figure 6:**
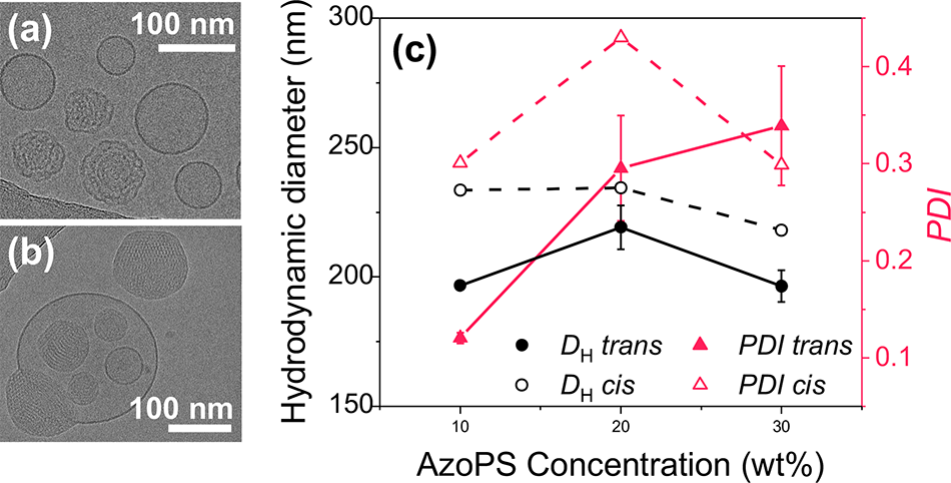
Change in particle size, shape, and polydispersity of MO-AzoPS-water dispersions on isomerization. TEM micrographs show the retention of spherical particles, with some containing internal order in MO dispersions containing 20 wt % initial water and 10 wt % of either (a) C_6_AzoC_4_E_4_ or (b) C_8_AzoC_8_E_4_, where more complex multi-vesicle structures also form. (c) Hydrodynamic diameter and polydispersity index (PDI) for a dispersion of MO, water (20 wt %), and varying concentration of AzoPS (C_6_AzoC_4_E_4_) in the *trans* and *cis* isomers. Note that there are no error bars for *cis* samples as only one measurement was taken for each sample to avoid reverse isomerization under the light beam.

The retention of internal order on isomerization was visible in TEM micrographs (Figure 6a,b); however, these also displayed heterogeneity in the particle structures. The micrographs show a mixture of vesicles, ordered LLC particles, and, for dispersions containing C_8_AzoC_8_E_4_, more complex, multi-particle assemblies consisting of multiple, ordered cubosome regions within a vesicle shell. Further optimization of the preparation method for these systems may be needed to form a homogenous array of cubosome particles, as has been achieved in the literature previously.^68^

DLS studies showed that the particle size increased on isomerization of the AzoPS (Figure 6c), resulting in hydrodynamic diameters mostly between 211 and 257 nm (see SI, Table S11). As in the *trans* state, an outlying diameter of 571 nm was observed for particles with 30 wt % C_8_AzoC_8_E_4_. The *cis* isomer of pure azobenzene has a larger dipole moment than the *trans*, resulting in an increase in the hydrophilicity of the AzoPS molecules on isomerization.^35^ The increased interaction between the molecules and the surrounding water may lead to swelling at the particle surface, which may explain the observed increase in hydrodynamic diameter. It is worth noting, however, that this swelling has no effect on the ordered, cubic LLC regions, in which there is a contraction of the lattice, as discussed above. In the *trans* state, the polydispersity showed a high dependence on the concentration of AzoPS within the LLCs. For low concentration samples (10 wt %), photoisomerization resulted in a large increase in polydispersity, compared to the *trans* state (Figure 6c). The formation of a PSS upon irradiation could lead to heterogeneity in the interaction between different particles and the surrounding water, resulting in a heterogeneity in the particle size. In contrast, for dispersions containing higher concentrations of AzoPS (30 wt %), this effect is masked by the high initial polydispersity for *trans* dispersions, leading to an insignificant change on isomerization.

### Light-Induced Release from MO-AzoPS Dispersions

**Figure 7:**
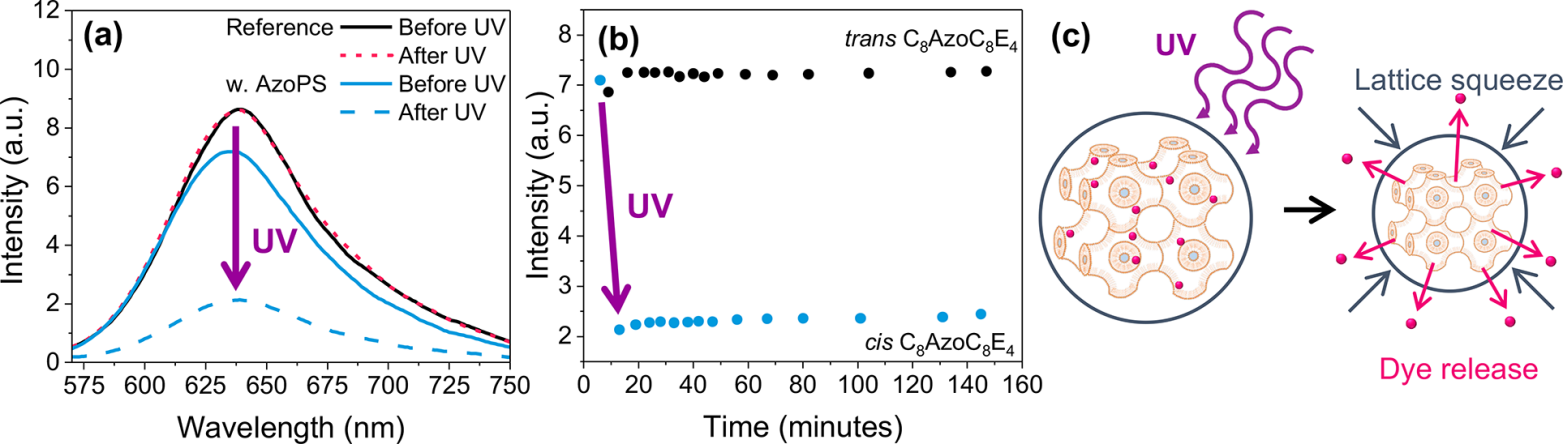
Release of Nile Red from MO-AzoPS dispersions. (a) Fluorescence spectrum (λ_ex_ = 550 nm) of Nile Red in dispersions of MO-water (20 wt %) and MO-C_8_AzoC_8_E_4_ (30 wt %)-water (20 wt), before and after irradiation with UV light (3 min). (b) Emission intensity (λ_em_ = 640 nm) as a function of time after dispersion, for dispersions with AzoPS in the *cis* (blue) and *trans* (black) states. (c) Schematic diagram showing the dye-release mechanism following lattice squeeze on irradiation with UV light.

The correlation between the change in lattice parameter upon irradiation and the ability of the cubosomes to retain and release guest molecules was next investigated. Nile Red was used as the guest molecule, a hydrophobic dye that exhibits a high fluorescence intensity when present in a lipid phase but significantly lower fluorescence in water.^69^ This allows the location of the dye, either in the lipid-like amphiphile bilayer or the surrounding aqueous dispersion phase, to be monitored from its emission spectrum. A reference dispersion was made by mixing Nile Red (0.03 wt %) into an MO-water (20 wt %) bulk mixture before homogenizing into a dispersion. Following excitation at 550 nm, which avoids inducing unwanted isomerization, a fluorescence peak was observed at 640 nm in the emission spectrum. This reference spectrum showed no change on irradiation of the dispersion using UV light for 3 min (Figure 7a). This was compared with the dispersion that showed the greatest change in the lattice parameter on isomerization, MO-C_8_AzoC_8_E_4_ (30 wt %)-water (20 wt %). For this sample, the fluorescence intensity at 640 nm decreased by 72% on irradiation with UV light, under identical conditions (Figure 7a). This indicates that Nile Red is released from the lipid LLC matrix into the aqueous phase as a result of the contraction of the cubic phase, which can be thought of as squeezing the dye from the amphiphile bilayer (Figure 7c).

The change in the fluorescence intensity with time after isomerization was also tracked and compared to an identical dispersion with the AzoPS kept in the *trans* state, through storage in the dark. Both samples retained a fluorescence peak of roughly the same intensity over the course of 3 h, indicating minimal diffusion of the dye out into the aqueous phase within this period (Figure 7b). This shows that stimulated release using UV irradiation is significant in comparison to the gradual diffusive release from these systems on the time scale of hours, within which thermal relaxation of the *cis* state back to the *trans* is not a concern. Structural control of the LLC therefore allows rapid, stimuli-responsive release in comparison to diffusion from the particles in their unirradiated state.

## Conclusions

In summary, we have designed light-responsive cubosomes that exhibit a swell-squeeze mechanism to enable triggerable release of entrapped payload. First, light-responsive AzoPS molecules were combined with MO and water to form bulk LLC mesophases whose structure depends on the chain length of the AzoPS, with C_6_AzoC_4_E_4_ and C_8_AzoC_8_E_4_ forming hexagonal and lamellar phases, respectively. Bulk LLCs were then homogenized to form stable dispersions of particles ∼200 nm in diameter. An internal inverse bicontinuous primitive cubic LLC phase was observed using SAXS and cryo-TEM across the composition (10–40 wt % initial water, 10–30 wt % C_6_AzoC_4_E_4_ or C_8_AzoC_8_E_4_) and temperature (25–55 °C) range tested. Notably, the cubic lattice parameter is highly sensitive to the AzoPS concentration: a higher loading leads to swelling of the cubic lattice, offering a method to tune the nanoscale structure. However, the stability of the cubic LLC phase decreased at higher AzoPS concentrations, suggesting that there is a sweet spot to be found between tunability and stability. Upon UV irradiation, AzoPS molecules within the cubosome structure isomerized rapidly between *trans* and *cis* states, leading to a small increase in particle size in most samples but retention of the internal inverse bicontinuous primitive cubic phase across the composition range. However, photoisomerization leads to squeezing of the cubic lattice, resulting in a corresponding decrease in the lattice parameter. This squeeze mechanism was successfully harnessed to enable phototriggerable release of trapped Nile Red guest molecules from the cubosome structure into the aqueous phase. It is thought that the “bending” of the AzoPS tail on isomerization leads to an increase in the tail volume and thus the spontaneous curvature of the amphiphile bilayer in the cubic LLC. This acts to contract the lattice, which effectively squeezes the hydrophobic dye out of the LLC matrix in a manner that is markedly faster than release due to diffusion.

With view to application, ordered LLC nanoparticles are promising candidates for the next-generation of nanodelivery devices for drugs, catalysts, or other active molecules. Triggerable release of the entrapped payload further directs delivery, which can improve selectivity and, notably for anti-cancer treatments, reduce drug toxicity to surrounding normal tissue. This proof-of-concept work has shown that cubosomes can be built containing light-responsive AzoPS, swelled (using composition) to allow design to encapsulate a variety of different payloads and subsequently squeezed (using photoisomerization) to induce release. Further work is needed to probe how this swell-squeeze mechanism can be exploited to tune the release of a greater variety of guest molecules with different hydrophilicities. Looking toward clinical application, red-shifting the isomerization wavelength from the UV to infra-red regions, using further functionalization of the azobenzene, is a vital step toward improving tissue penetration and paving the way toward light-triggerable release *in vivo*.

## Supporting Information Available

Materials and experimental methods; details for the synthesis and characterization of C_6_AzoC_4_E_4_ and C_8_AzoC_8_E_4_ using NMR spectroscopy, FTIR, mass spectrometry, and percentage yields; AzoPS critical micelles concentrations; MO-water bulk LLC characterization; calculations to estimate MO, C_6_AzoC_4_E_4_, and C_8_AzoC_8_E_4_ geometries; DLS results for LLC particle size and stability after 10 months; photographs to show dispersion stability after 10 months; cryo-TEM micrograph for MO-C_8_AzoC_8_E_4_-water dispersion; additional SAXS data and analysis for dispersions in the *trans* state, Guinier size analysis, table of Bragg peak positions for phase assignment with temperature and composition, and calculations for the final water fraction in the nanoparticles; additional UV–vis absorption spectra, kinetics plots and results for AzoPS LLC dispersions on irradiation with UV and blue light, and thermal reversion of the *cis* form in the dark; additional SAXS data comparing isomerized dispersions; and DLS results for isomerized dispersions (PDF)

## Abbreviations

AzoPS: azobenzene photosurfactant
CPP: critical packing parameter
DLS: dynamic light scattering
LED: light-emitting diode
LLC: lyotropic liquid crystal
MO: monoolein
POM: polarized optical microscopy
SAXS: small-angle X-ray scattering
TEM: transmission electron microscopy
UV: ultra-violet
