# Supplementary material for: Light-Responsive Molecular Release from Cubosomes Using Swell-Squeeze Lattice Control
Source: J Am Chem Soc. 2022 Oct 12;144(42):19532–41. doi: 10.1021/jacs.2c08583 (PMC9619397; doi:10.1021/jacs.2c08583)
Supplement: Supplementary file 1 — ja2c08583_si_001.pdf [file ja2c08583_si_001.pdf]

Supporting Information

Light-responsive molecular release from cubosomes using swell-squeeze lattice control

Beatrice E. Jones,[a,b] *Elaine A. Kelly,*[a] *Nathan Cowieson,*[b] *Giorgio Divitini,*[a] *and Rachel C. Evans*[a]*

[a] Department of Materials Science & Metallurgy, University of Cambridge, 27 Charles Babbage Road, CB3 0FS, United Kingdom

[b] Diamond Light Source, Harwell Science and Innovation Campus, Didcot, Oxfordshire, United Kingdom

* Corresponding Author: rce26@cam.ac.uk

TABLE OF CONTENTS

1 EXPERIMENTAL METHODS 3

1.1 MATERIALS 3

1.2 PREPARATION OF BULK LLCS AND DISPERSIONS 3

1.3 SAMPLE IRRADIATION 3

1.4 SMALL-ANGLE X-RAY SCATTERING 3

1.5 POLARIZED OPTICAL MICROSCOPY 4

1.6 DYNAMIC LIGHT SCATTERING 4

1.7 CRYO-TRANSMISSION ELECTRON MICROSCOPY 4

1.8 UV-VIS ABSORPTION SPECTROSCOPY 5

1.9 RELEASE STUDIES USING NILE RED 5

2 SYNTHESIS AND CHARACTERIZATION OF AZOBENZENE PHOTOSURFACTANTS (AZOPS) 5

2.1 MATERIALS 5

2.2 METHODS 5

2.3 STRUCTURAL CHARACTERIZATION 7

3 CRITICAL MICELLE CONCENTRATIONS FOR AZOPS 8

4 CHARACTERIZATION OF MO-WATER BULK LLCS 8

5 ESTIMATING AMPHIPHILE GEOMETRIES 9

6 SIZE AND STABILITY OF LLC DISPERSIONS 11

7 CRYO-TEM MICROSCOPY 14

8 SMALL-ANGLE X-RAY SCATTERING FOR DISPERSIONS WITH AZOPS IN THE *TRANS* STATE 14

9 UV-VIS ABSORPTION SPECTRA 21

10 AZOPS ISOMERIZATION KINETICS 22

11 SAXS DATA FOR ISOMERIZED DISPERSIONS 25

12 DYNAMIC LIGHT SCATTERING FOR ISOMERIZED DISPERSIONS 27

13 REFERENCES 28

1 Experimental Methods

1.1 Materials

4-hexyl-4'-(mono-tetraethylene glycol) butoxy azobenzene ($C_6AzoC_4E_4$) and 4-octyl-4'-(mono-tetraethylene glycol) octoxy azobenzene ($C_8AzoC_8E_4$) were synthesized using previously reported method.[1,2] Full details of the synthetic approach and structural characterization are given below (Section 2). 1-Oleoyl-*rac*-glycerol (monoolein, MO, ≥99%), Nile Red and Pluronic F-127 were purchased from Sigma Aldrich and used as received. Water was obtained from a Millipore Simak 2 water purification system.

1.2 Preparation of Bulk LLCs and Dispersions

Bulk LLC phases were made by heating constituent components (MO and water, with either $C_6AzoOC_4E_4$, $C_8AzoOC_8E_4$ or no AzoPS) to 60 °C whilst stirring, until well mixed. The AzoPS concentration was varied with respect to MO (0-30 wt%) and initial water concentration was varied with respect to MO + AzoPS (10-40 wt%). To form dispersions, bulk MO-based LLCs were added to a solution of Pluronic F-127 (0.3 wt%) in water at a concentration of 5 wt%. Samples were homogenized with a SciQuip Basic motorized homogenizer at 15,000 rpm for 10-30 minutes, until visibly uniform.

1.3 Sample Irradiation

For photoisomerization, samples were irradiated using LEDs, of either UV (irradiance = 6.00 mW cm^{-2}, wavelength = 365 nm) or blue (5.16 mW cm^{-2}, 455 nm) light. Samples were irradiated for at least 10 minutes, which was shown to be sufficient to reach the *cis*-dominant PSS by UV-Vis absorption spectroscopy. For SAXS studies, isomerization was further confirmed by taking the UV-Vis absorption spectrum following irradiation on a NanoDrop 1000 spectrophotometer (Thermo Scientific).

1.4 Small-angle X-ray scattering

SAXS measurements were performed at the BioSAXS beamline B21, Diamond Light Source.[3] A fixed sample-to-detector distance (4.014 m) and X-ray beam energy (12.4 keV) gave an available q range of 0.0045-0.34 $Å^{-1}$, where q is the scattering vector given by $q = 4\pi/\lambda \sin(\theta/2)$, λ = X-ray wavelength and θ = elastic scattering angle. 30 frames were collected for each sample at 1 frame s^{-1}. Bulk LLC phases were either manually injected into polyimide capillaries or loaded into 3D-printed custom 'lollysticks' and enclosed using Kapton tape before measurement. Dispersions were loaded into PCR well plates and stored at 25 °C,

before being delivered to a quartz capillary for measurement using the BioSAXS robot. Samples were moved at 1 µL s^{-1} through the beam during measurement to avoid beam damage. 2D diffraction patterns were radially averaged and integrated to get 1D data. The water background was then subtracted using the ScÅtter software.[4] Unless otherwise stated, results are given for samples at 25 °C, before temperature ramp. All temperature-dependent data are given for samples that were equilibrated for 5-10 minutes until no further changes to the scattering patterns were observed. For lattice parameter calculations, peak positions were found by subtracting the background and fitting the data to Gaussian, Lorenzian or Gaussian-Lorentzian functions using Origin(Pro), Version 2021b, OriginLab Corporation. For phase identification in temperature scans, peak positions were found using the Find Peaks tool in Origin(Pro).

1.5 Polarized Optical Microscopy

Most POM images were taken using a Leica EC4 camera fitted to an Olympus BHM microscope. For the micrographs in Figures S1a and S1c, an Axiocam 208c was fitted to a Zeiss Primostar 3. Samples in the *trans* state were placed between a glass slide and cover slip and pressed to ensure they were sufficiently thin to allow light transmission. All samples were measured at room temperature under crossed polars.

1.6 Dynamic Light Scattering

Dispersions were diluted by a factor of 4000, using triple-filtered water (0.2 µm). Measurements were taken using a Malvern Instruments Zetasizer Nano ZS in backscatter mode (173º), using a He-Ne laser emitting at 633 nm, and an equilibration time of 60 s. Cumulant analysis of the correlation function was used to determine the mean Z-average hydrodynamic diameter and the polydispersity of each sample.[5] The fit report from the instrument was used to disregard poorly fitted results. Results were averaged over 3 runs of 14 scans each, with the error given by the standard deviation. For AzoPS dispersions in the *cis* state, only the first run was used to avoid effects of reverse isomerization from the light beam.

1.7 Cryo-Transmission Electron Microscopy

Samples were vitrified in a controlled environment using a ThermoFisher Vitrobot. *Cis* samples were irradiated with UV light for 10 minutes prior to vitrification. A volume (2.5 µL) of sample was deposited onto a Quantfoil 1.2/1.3 holey carbon film. Samples were blotted for 2.5 s before plunging into liquid ethane. Vitrified specimens were stored in liquid nitrogen (-196 °C)

before imaging. The microscope used was a ThermoFisher Krios G3i with an acceleration voltage of 300 kV and images were captured on a Falcon4 camera.

1.8 UV-Vis Absorption Spectroscopy

Dispersions and AzoPS samples were diluted in water to reduce the absorbance and prevent detector saturation. Spectra were recorded on an DS5 spectrometer (Edinburgh Instruments) with a slit width of 4 nm and a scan speed of 200 nm min^{-1}, using quartz cuvettes with a 10 mm path length.

1.9 Release Studies using Nile Red

Emission spectra were recorded on a Horiba Jobin Yvon Flurolog®-3 Spectrofluorometer using an excitation wavelength of 550 nm and slit widths of 1 nm. Spectra were recorded between 570-750 nm (increment = 1 nm, integration time = 0.5 s) and smoothed using the Savitzky-Golay method (window = 20 points). Bulk phases were prepared as before, containing MO-$C_8AzoC_8E_4$ (30 wt%)-water (20 wt%) and MO- water (20 wt%). Nile Red (0.03 w/w%) was dissolved in the bulk phase before homogenization. Dispersions were diluted in water by a factor of 500 to avoid detector saturation. For both the AzoPS and reference dispersions, two dilute samples were prepared and fluorescence spectra taken. One of the samples was then UV-irradiated for 5 minutes, the other was kept in the dark. Sequential fluorescence spectra of both samples were then taken over the course of 3 hours.

2 Synthesis and characterization of azobenzene photosurfactants (AzoPS)

2.1 Materials

Sodium nitrite ($NaNO_3$, ≥99.0%), anhydrous potassium carbonate (≥99.0%), potassium iodide (KI, 99.0%), sodium hydride (60% w/w in mineral oil), tetraethylene glycol (99%), hydrochloric acid (HCl, conc. 37%), ethanol (HPLC grade), tetrahydrofuran (THF, HPLC grade), dichloromethane (DCM) and acetone were purchased from Sigma Aldrich. 4-hexylaniline (90%) was purchased from ChemCruz. Sodium hydroxide (NaOH, ≥97%) and chloroform (>99%) were purchased from Fisher Scientific and phenol (>99.0%) was purchased from BDH Chemical Ltd. England. 4-octylaniline (99%) and 1,4-dibromobutane (>98%) were purchased from Alfa Aesar. All reagents and solvents were used as received unless otherwise stated.

2.2 Methods

Two different AzoPS structures were synthesized, both containing a tetraethylene glycol head group, but with differing alkyl spacer (of length *m* = 4 or 8) or alkyl tail (*n* = 6 or 8) lengths,

subsequently referred to as $C_6AzoC_4E_4$ and $C_8AzoC_8E_4$. Synthesis of the AzoPS was achieved through three steps: (1) the preparation of a hydroxyl precursor, 4-alkyl-4′-hydroxylazobenzene; (2) modification of the above to a bromoprecursor, 4-alkyl-4′-(2-bromo)alkyloxy azobenzene, via an S_N2 reaction; (3) addition of the neutral tetraethylene glycol head group to yield the final product.

2.2.1 Synthesis of C_mAzoOH (4-alkyl-4'hydroxyl azobenzene, where alkyl = hexyl or octyl for *m* = 6 and 8, respectively)

4-alkylaniline (20 mmol; 4-hexylaniline: 3.85 mL; 4-octylaniline: 4.57 mL) was dissolved in an acetone: water mixture (1:1, 50 mL) and HCl (37.5%, 0.048 mol, 4 mL) added. The resulting orange solution was placed in an ice bath and kept at 0 °C. Sodium nitrite (20 mmol, 1.38 g) was dissolved in distilled water (20 mL), cooled (1-2 °C) and added dropwise to the acidic alkylaniline solution. The orange solution was left to stir over ice for 5 min, to allow for diazonium salt formation. The solution was placed in the freezer until required. Separately, NaOH (20 mmol, 0.80 g), Na_2CO_3 (20 mmol, 2.12 g) and phenol (20 mmol, 1.88 g) were dissolved in distilled water (50 mL) and cooled until the solution temperature reached 1-3 °C. To the basic solution, the acidic diazonium salt solution was added dropwise, maintaining a temperature < 8 °C. An aqueous NaOH solution (6.5 mL, 2.5 mmol L^{-1}) was added dropwise to the basic phenol solution along with the acidic diazonium salt solution, to maintain a final pH of 10. The resulting yellow precipitate was filtered and washed with cold water. The product C_mAzoOH was recovered in the form of a yellow powder.

2.2.2 Synthesis of bromoprecursor C_mAzoOC$_n$Br (4-alkyl-4'(4/8-bromo) alkoxy azobenzene, where alkyl = hexyl or octyl for *m* = 6 and 8, respectively. 4/8 and alkoxy = butoxy or octoxy for *n* = 4 or 8)

C_mAzoOH (1 eq., 10 mmol, 2.82 g for C_6AzoOH; 3.00 g for C_8AzoOH) was dissolved in acetone (25 mL). 1,4-Dibromobutane or 1,8-dibromooctane (2 eq., 20 mmol, 2.39 or 3.68 mL), K_2CO_3 (2 eq., 20 mmol, 2.76 g) and KI (0.1 eq., 1 mmol, 0.17 g) were added to the reaction flask and the resulting dark red solution was stirred under reflux at 65 °C for 2 days with monitoring via thin layer chromatography (TLC, 9:1 cyclohexane: ethyl acetate). Acetone was removed via rotary evaporation and the product was dissolved in DCM (20 mL) and washed with water several times to remove the co-salt. DCM was removed via rotary evaporation and the product was recovered as an orange solid upon recrystallization from ethanol.

2.2.3 Synthesis of C_mAzoO$C_n$$E_4$ (4-alkyl-4'-(mono-tetraethylene glycol) alkoxy azobenzene, replace alkyl with hexyl or octyl for *m* = 6 and 8 respectively, and alkoxy = butoxy or octoxy for *n* = 4 or 8)

Tetraethylene glycol (5 eq., 28.1 mmol, 5.41 mL) was dried using molecular sieves. Dry tetraethylene glycol was then dissolved in dry THF (25 mL) in a round-bottomed flask (50 mL) under an inert N_2 atmosphere. To this NaH (60% in oil (w/w), 1.5 eq., 8.4 mmol, 0.346 g) was added and left to stir for 1 h under N_2. C_mAzoOC_nBr (1.0 eq., 5.6 mmol) was dissolved in dry THF (10 mL) and added to the reaction flask. The resulting red solution was refluxed at 65 °C for 24 h. Acetone was removed via rotary evaporation and the resulting red oil dissolved in dichloromethane (20 mL) and washed with water. The DCM layer was dried, filtered and the solvent was removed via rotary evaporation. Excess bromoprecursor was removed using a silica plug and washing with chloroform. The product was recovered as a red oil.

2.3 Structural Characterization

2.3.1 C_6Azo$C_4$$E_4$

^{1}H NMR: ($CDCl_3$, 400 MHz, 25 °C): δ = 0.9 (t, *J* = 8 Hz, 3H), 1.22 (d, 2H), 1.33 (m, 6H), 1.66-1.9 (m, 6H), 2.69 (t, *J* = 8 Hz, 2H), 3.63 (m, 16H), 4.09 (t, J = 7 Hz, 2H), 7 (d, J = 8.88 Hz, 2H), 7.3 (d, J = 8.16 Hz, 2H), 7.8 (d, J = 8.2 Hz, 2H), 7.89 (d, J = 8.8 Hz, 2H) ppm.

^{13}C NMR: ($CDCl_3$, 600 MHz, 25 °C): δ = 14.1, 22.6, 26.1, 28.9, 30.9, 31.3, 31.7, 35.4, 61.7, 68, 70-70.9 (7 peaks), 72.65, 114.68, 122.51, 124.55, 129.03, 145.8, 146.9, 151, 161.4 ppm

FTIR: *v* = 3175 (br.), 2959 (w), 2926 (m), 2873 (w), 1603 (s), 1490 (m), 1256 (s), 1131 (m) cm^{-1}.

Mass Spectrometry (CH_3Cl, *m*/*z*: APCI$^+$): Exact mass calculated: 530.3356. Exact mass obtained: 531.3442 $[M+H]^+$

Yield: 39%

2.3.2 C_8Azo$C_8$$E_4$

^{1}H NMR: ($CDCl_3$, 400 MHz, 25 °C): δ = 0.84 (t, *J* = 8 Hz, 3H), 1.26–1.54 (m, 24H), 1.78 (m, 2H), 2.63 (t, *J* = 8 Hz, 2H), 3.64 (m, 16 H), 3.99 (t, *J* = 7 Hz, 2H), 6.95 (d, *J* = 9 Hz, 2H), 7.25 (d, *J* = 8.32 Hz, 2H), 7.75 (d, *J* = 8.36 Hz, 2H), 7.84 (d, *J* = 8.96 Hz, 2H) ppm.

^{13}C NMR: ($CDCl_3$, 600 MHz, 25 °C): δ = 14.06, 22.61, 25.93, 29.2, 29.3, 29.2, 29.3, 29.3, 29.3, 30.9, 31.3, 31.8, 33.6, 35.8, 61.5, 68.3, 70.2-70 (7 peaks), 72.8, 114.6, 122.5, 124.5, 129, 145.8, 146.9, 151, 161.1 ppm.

FTIR: *v* = 3169 (br.), 2971 (w), 2928 (m), 2867 (w), 1594 (s), 1498 (s), 1247 (s), 1139 (s) cm^{-1}.

Mass Spectrometry (CH_3Cl, *m*/*z*: APCI$^+$): Exact mass calculated: 614.4295 [M]. Exact mass obtained: 615.439 $[M+H]^+$.

Yield: 47%

3 Critical micelle concentrations for AzoPS

The critical micelle concentrations (CMCs) for both AzoPS structures in both the *trans* and *cis* isomers have been measured and reported previously, using both surface tensiometry (ST) and dynamic light scattering (DLS).[1]

Table S1. Critical micelle concentrations (CMCs) of the *trans* and *cis* isomers of the AzoPS determined using dynamic light scattering (DLS) and surface tensiometry (ST), as reported in the literature.[1] The associated errors are given from the standard deviation of three measurements.

	**CMC by ST (μM)**		**CMC by DLS (μM)**	
	trans isomer	*cis* isomer	*trans* isomer	*cis* isomer
$C_6AzoC_4E_4$	37.1 ± 6.3	29.5 ± 1.8	22.9 ± 4.2	23.7 ± 5
$C_8AzoC_8E_4$	108.6 ± 3.1	125.9 ± 2.3	102.7 ± 33.9	12.4 ± 1.2

4 Characterization of MO-water bulk LLCs

Bulk LLC phases in reference samples of MO and water were characterized using a combination of SAXS and POM. At a concentration of 20 wt% water, MO formed a lamellar LLC, visible as diffraction peaks with a ratio of 1:2 in the SAXS data (Figure 1) and an oily streak pattern using POM (Figure S1b). To further confirm LLC phase behavior POM micrographs were taken at increasing water concentrations. The expected crystal → lamellar → cubic transitions were observed on increasing the water concentration (Figure S1). Further confirmation of standard MO phase behaviour in the excess-water regime was investigated using SAXS. A capillary was coated with MO, filled with water and left to equilibrate before measurement. The bicontinuous diamond cubic (*Pn3m*) phase was observed, as expected. The lattice parameter of 130 Å, was comparable to literature values for the fully hydrated phase of 128.6 Å.[6]

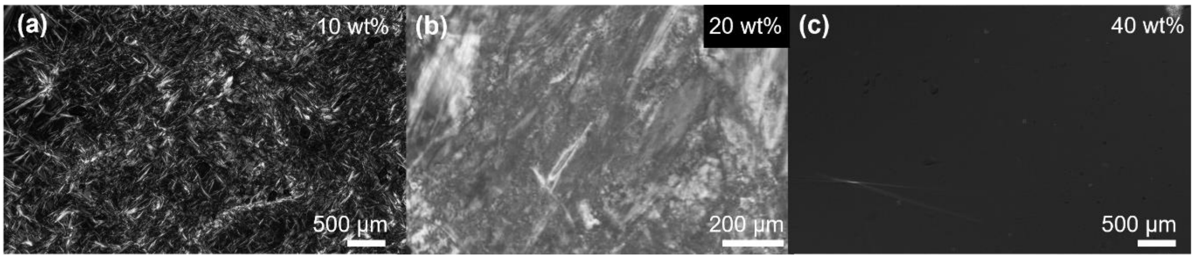

Figure S1. Formation of LLC phases in MO at increasing water concentrations (10–40 wt%) at 25 °C. POM micrographs show: sharp lines characteristic of the crystalline phase (10 wt%); oily streak pattern characteristic of lamellar LLC (20 wt%); and darkness, indicative of formation of the isotropic cubic LLC phase (40 wt%).

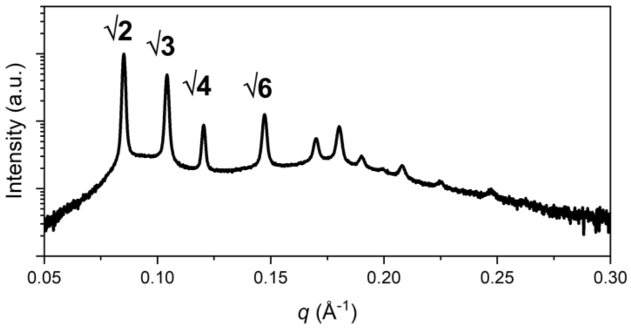

Figure S2. SAXS curve for MO in excess water (T = 20 °C), showing the formation of the bicontinuous diamond cubic (*Pn3m*) phase from Bragg peaks in the ratio √2:√3:√4:√6. Error bars have been removed for clarity.

5 Estimating amphiphile geometries

The critical packing parameter (*CPP*) for an amphiphile can be used to predict the lyotropic liquid crystal phase it is likely to form due to the spontaneous curvature favoured by the self-assembled amphiphiles.[1] The *CPP* is defined as: $CPP = v/a_0l_c$, where v is the volume of the hydrophobic tail, a_0 is the hydrophilic head group area and l_c is the length of the hydrophobic chain.[7] To calculate the packing parameter for AzoPS, v and l_c were determined using the empirical volume additivity rule of Traube:[8]

$$v = \sum v_i \quad \text{Eq. S1}$$

$$l_c = \sum l_{ci} \quad \text{Eq. S2}$$

where v_i and l_{ci} are the contributions of the *i*th components for the tail volume and length, respectively.

The Tanford equations are commonly used to calculate the lengths and volumes of alkyl chain of surfactants[9] and were used to obtain the contributions of the alkyl chain of AzoPS.

$$v = 26.9 \times m + 27.4 \quad \text{Eq. S3}$$

$$l_c = 1.265 \times m + 1.5 \quad \text{Eq. S4}$$

where m is the number of $-CH_2-$ carbons in the carbon chain. Further contributions to v and l_c were found using literature values from various methods as summarized in Table S2. The contributions were added to give estimates for v, a_0 and l_c for the two different AzoPS structures in the *trans* isomeric state (Table S3). These were used to calculate the *CPP* and

the ratio of head-group area to tail length (a_0 / l_c), which are both important considerations to determine expected LLC phase formation. These were compared with literature values for MO.[10]

Table S2. Contribution of volumes, lengths and head-group areas to calculate the critical packing parameter, *CPP*, for AzoPS in the *trans* isomeric state using the additivity rule.

Component	Value	Method
Alkyl chain volume (nm^3)	0.0269*m* + 0.0274	Tanford equations[9]
trans azobenzene volume (nm^3)	0.176	MOPAC calculations, van der Waals volume[11]
Oxy-group volume (nm^3)	9.1×10^{-3}	*Ab initio* calculations, van der Waals volume[8]
Alkyl chain length (nm)	0.1265*m* + 0.15	Tanford equations[9]
trans azobenzene length (nm)	0.9	X-ray analysis[12]
Oxy-group length (nm)	0.28	DFT calculations[13]
E_4 head-group area (nm^2)	0.46	Surface tension study of $C_{12}E_4$ at 25 °C[14]

Table S3. Amphiphile geometrical parameters as calculated for AzoPS in the *trans* state and obtained from the literature for MO. The volume of the hydrophobic tail, *v*; hydrophilic head group area, a_0; and length of the hydrophobic chain, l_c, contribute to the critical packing parameter, *CPP*, and ratio of head-area to tail length (a_0 / l_c). Note that the data for MO is calculated for 45 °C, compared to 25 °C for $C_6AzoC_4E_4$ and $C_8AzoC_8E_4$.

**Amphiphile**	***v*** **(nm³)**	a_0 **(nm²)**	l_c **(nm)**	***CPP***	a_0 / l_c **(nm)**
MO	0.53[10]	0.25[10]	1.85[10]	1.16[10]	0.14
$C_6AzoC_4E_4$	0.48	0.46	2.60	0.40	0.18
$C_8AzoC_8E_4$	0.64	0.46	3.35	0.42	0.14

6 Size and stability of LLC dispersions

Table S4. Particle size distribution within MO-AzoPS-water LLC dispersions (T = 25 °C). Changes in the Z-average hydrodynamic diameter, D_H, and polydispersity index, *PDI*, with varying composition and tail length of AzoPS, as measured using DLS one month after preparation.

	**AzoPS (wt%)**	**Initial Water (wt%)**	D_H **(nm)**		**PDI**	
**MO-water**	0	10	184 ± 4		0.31 ± 0.03	
	0	20	152 ± 2		0.23 ± 0.01	
	0	30	175 ± 2		0.34 ± 0.01	
	0	40	130 ± 1		0.18 ± 0.03	
**AzoPS**			$C_6AzoC_4E_4$	$C_8AzoC_8E_4$	$C_6AzoC_4E_4$	$C_8AzoC_8E_4$
	10	10	159 ± 4	192 ± 1	0.13 ± 0.03	0.34 ± 0.01
	10	20	197 ± 2	179 ± 3	0.12 ± 0.01	0.15 ± 0.01
	10	30	192 ± 2	176 ± 4	0.12 ± 0.01	0.14 ± 0.01
	10	40	194 ± 5	186 ± 4	0.14 ± 0.02	0.27 ± 0.01
	20	20	219 ± 8	191 ± 3	0.30 ± 0.05	0.12 ± 0.01
	30	20	196 ± 6	468 ± 7	0.34 ± 0.06	0.32 ± 0.07

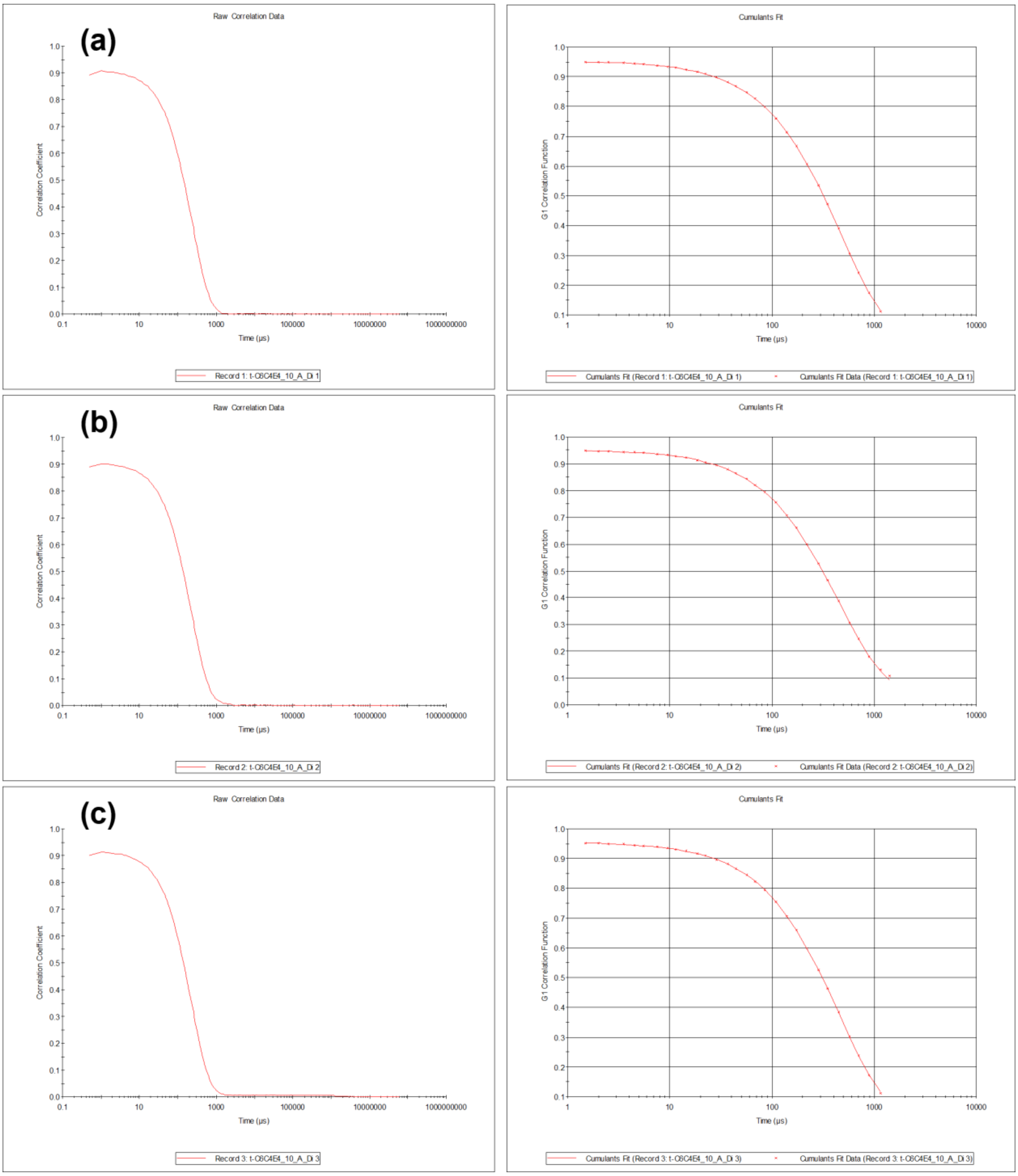

Figure S3. Example plots for the correlation data and cumulants fits used to calculate the Z-average hydrodynamic diameter, D_H, and polydispersity index, *PDI*, for a dispersion of MO-C_6AzoC_4E_4 (10 wt%)-water (10 wt%).

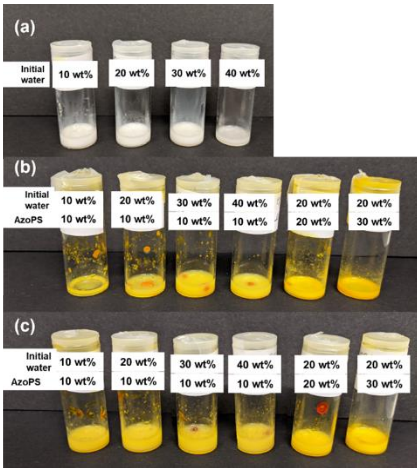

Figure S4. Stability of LLC dispersions after storage in the dark for 10 months. Images show (a) MO-water dispersions with increasing initial water concentration; and MO-AzoPS-water dispersions with varying initial water (10-40 wt%, at constant AzoPS concentration, 10 wt%) and AzoPS (10-30 wt%, at constant initial water concentration, 20 wt%) for (b) $C_6AzoC_4E_4$ and (c) $C_8AzoC_8E_4$. Whilst there are some agglomerates at the side of the vials, the cloudy nature of the samples shows retention of some nanoparticles in dispersion. Complete phase separation occurred in none of the samples.

Table S5. Particle size distribution within MO-AzoPS-water LLC dispersions (T = 25 °C) after storage in the dark for 10 months. Changes in the hydrodynamic diameter, D_H, and polydispersity index, *PDI*, with varying composition and tail length of AzoPS as measured using DLS.

	**AzoPS (wt%)**	**Initial Water (wt%)**	**D_H (nm)**		**PDI**	
**MO-water**	0	10	119 ± 1		0.42 ± 0.04	
	0	20	115 ± 3		0.26 ± 0.02	
	0	30	95 ± 2		0.24 ± 0.03	
	0	40	115 ± 1		0.33 ± 0.02	
**AzoPS**			**$C_6AzoC_4E_4$**	**$C_8AzoC_8E_4$**	**$C_6AzoC_4E_4$**	**$C_8AzoC_8E_4$**
	10	10	80 ± 3	104 ± 1	0.19 ± 0.01	0.38 ± 0.02
	10	20	100 ± 3	117 ± 1	0.49 ± 0.04	0.41 ± 0.01
	10	30	103 ± 3	88 ± 1	0.38 ± 0.02	0.26 ± 0.01
	10	40	90 ± 1	114 ± 5	0.29 ± 0.03	0.60 ± 0.10
	20	20	220 ± 7	89 ± 2	0.35 ± 0.02	0.25 ± 0.01
	30	20	78 ± 3	173 ± 2	0.17 ± 0.01	0.27 ± 0.01

7 Cryo-TEM Microscopy

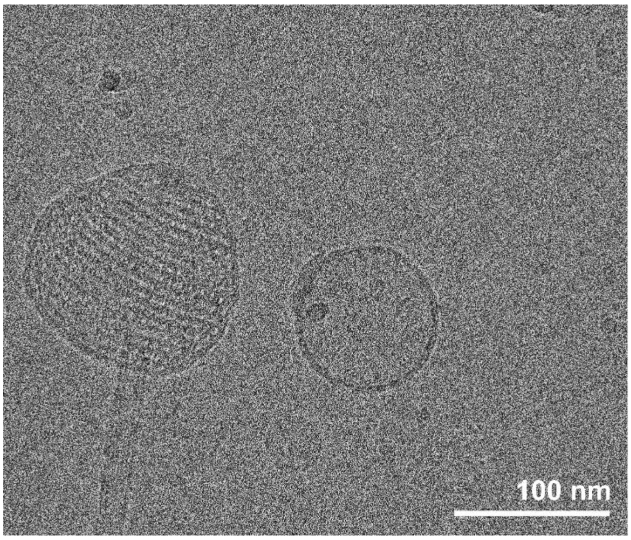

Figure S5. Cryo-TEM micrograph of dispersed particles of MO-$C_8AzoC_8E_4$ (10 wt%)-water (10 wt%). The particle on the left shows signs of internal order, whilst the particle on the right has a simple vesicle structure.

8 Small-angle X-ray scattering for dispersions with AzoPS in the *trans* state

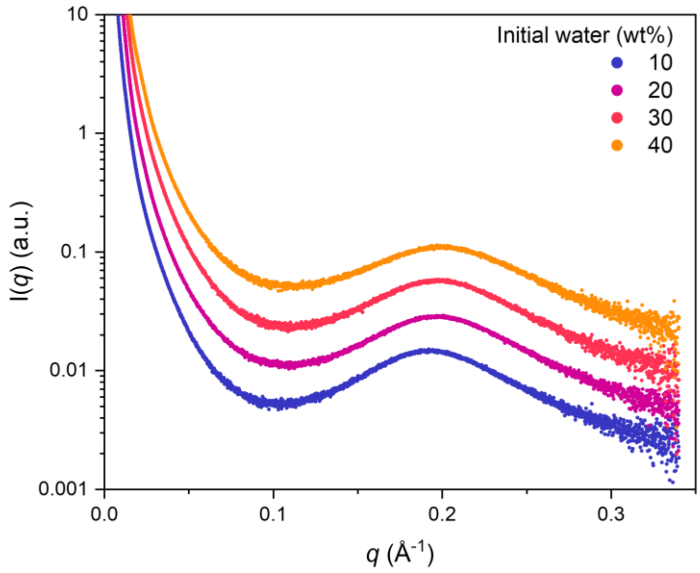

Figure S6. SAXS patterns for MO-water dispersions (T = 25 °C) of increasing initial water concentration, showing the retention of the broad peak at q = 0.2 $Å^{-1}$, which is characteristic of the vesicle bilayer packing. Error bars have been removed and curves offset for clarity.

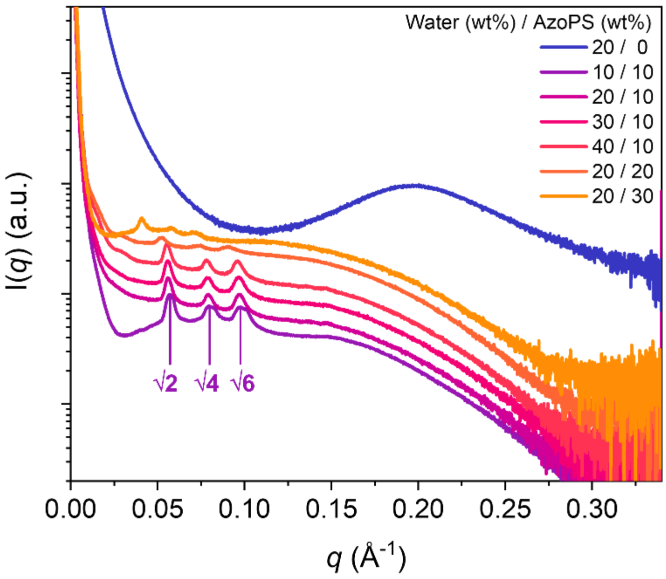

Figure S7. SAXS curves for dispersions of MO with varying concentration of initial water and $C_6AzoC_4E_4$ in the native, *trans* state (T = 25 °C). Bragg peaks in the ratio √2:√4:√6 show the formation of the inverse bicontinuous primitive cubic phase (*Im3m*) in samples containing $C_6AzoC_4E_4$. Error bars have been excluded and curves offset for clarity.

To estimate the particle size from the SAXS plots, the low *q* region of logI(*q*) vs. log*q* graphs were investigated. Here, the form factor P(*q*) can be approximated by a Gaussian curve, whose curvature is dependent on the particle size according to: [15]

$$\mathrm{I}(q) = I_0 e^{-\frac{R_G{}^2 q^2}{3}} \qquad \text{Eq. S5}$$

where I_0 is the extrapolated zero-angle intensity and R_G is the mean square radius of gyration. A Guinier plot, lnI(*q*) vs. q^2, for monodisperse particles shows a straight line with a slope of $-R_G/3$ up to the limit of $qR_G < 1.3$.[15] Guinier plots were used to determine the particle size in MO-water reference dispersions on increasing initial water concentration (Figure S8a) and in MO-$C_6AzoC_4E_4$-water dispersions on increasing AzoPS concentration (Figure S8b).

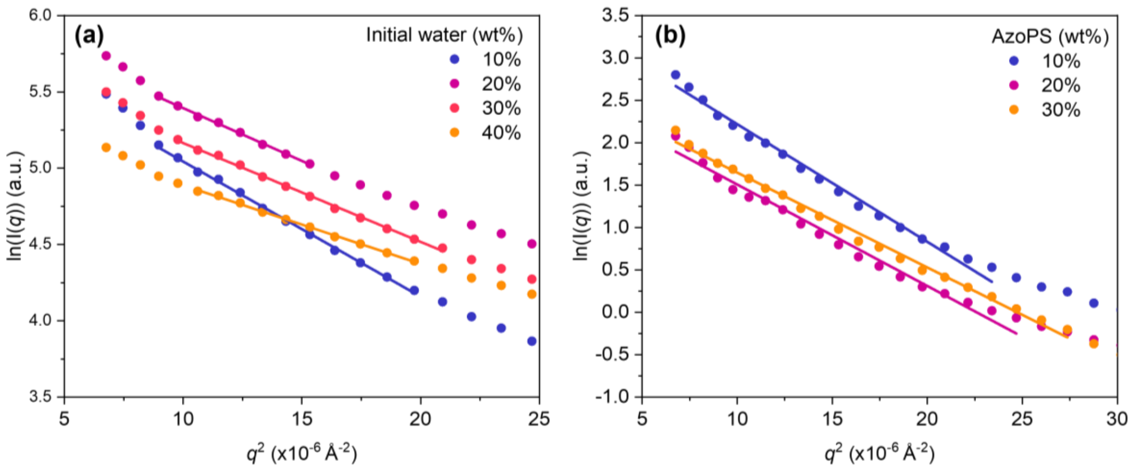

Figure S8. Guinier plots used to determine the size of dispersed **(a)** MO-water and **(b)** MO-$C_6AzoC_4E_4$-water particles with increasing concentrations of **(a)** initial water and **(b)** $C_6AzoC_4E_4$. Plots of ln(I(*q*)) vs. q^2 in the low *q* region for the SAXS data give straight-line plots whose gradient can be used to indicate the radius of gyration of the scattering particles.

Taking the particles to be spherical and of even density, the average particle, *D*, can be calculated from $D = (2\sqrt{5})/3\ R_G$.[16] The results from this calculation shown in Table S6 are comparable with the diameters obtained by DLS and cryo-TEM. The maximum value of q^2 that this analysis is valid up to, q^2_{max}, was calculated using the results for R_G. This showed that the straight-line regions used in this analysis lie beyond the range through which it is strictly accurate ($qR_G < 1.3$). This analysis can therefore only be used as a rough check that the scattering from the particle form factor agrees with the particle size determined by other techniques.

Table S6. Guinier size determination of dispersed MO-water and MO-$C_6AzoC_4E_4$-water particles. Calculated values for the scattering diameter, *D*, and theoretical maximum valid value of q^2 for this analysis, q^2_{max}, with increasing concentrations of initial water and $C_6AzoC_4E_4$.

	**AzoPS (wt%)**	**Initial Water (wt%)**	***D* (nm)**	**q^2_{max} (x10^{-6} Å^{-2})**
MO-water	0	10	133 ± 1	6.4
	0	20	118 ± 3	8.1
	0	30	114 ± 2	8.7
	0	40	102 ± 1	10.9
MO-$C_6AzoC_4E_4$-water	10	10	167 ± 2	4.1
	20	10	150 ± 1	5.0
	30	10	155 ± 3	4.7

Table S7. Peak positions for MO-AzoPS-water LLC dispersions of varying compositions and temperatures, with AzoPS in the native, *trans* state. The ratio of *q* peak positions of roughly 1:√2:√3 corresponds to √2:√4:√6, indicating the primitive cubic (*Im3m*) phase across this composition and temperature range.

AzoPS conc. (wt%)	Water conc. (wt%)	Temperature (°C)	$C_6AzoC_4E_4$						$C_8AzoC_8C_4$					
			q_1	q_2	q_3	q_1/q_1	q_2/q_1	q_3/q_1	q_1	q_2	q_3	q_1/q_1	q_2/q_1	q_3/q_1
10	10	25	0.05708	0.07984	0.09787	1.00	1.40	1.71	0.05708	0.07932	0.09748	1.00	1.39	1.71
		35	0.0605	0.08484	0.10353	1.00	1.40	1.71	0.05787	0.08142	0.10024	1.00	1.41	1.73
		45	0.06747	0.09511	0.11616	1.00	1.41	1.72	0.06142	0.08656	0.10603	1.00	1.41	1.73
		55	0.07392	0.10366	0.12722	1.00	1.40	1.72	0.06642	0.0934	0.11445	1.00	1.41	1.72
10	20	25	0.05589	0.07932	0.09708	1.00	1.42	1.74	0.05682	0.0805	0.09827	1.00	1.42	1.73
		35	0.05589	0.07932	0.09708	1.00	1.42	1.74	0.05682	0.08077	0.09892	1.00	1.42	1.74
		45	0.06063	0.08577	0.10458	1.00	1.41	1.72	0.06089	0.0855	0.10485	1.00	1.40	1.72
		55	0.06682	0.09366	0.11472	1.00	1.40	1.72	0.06734	0.09432	0.11577	1.00	1.40	1.72
10	30	25	0.05603	0.0784	0.09682	1.00	1.40	1.73	0.05655	0.07971	0.098	1.00	1.41	1.73
		35	0.05629	0.07866	0.09682	1.00	1.40	1.72	0.05642	0.07971	0.09774	1.00	1.41	1.73
		45	0.06142	0.08616	0.10577	1.00	1.40	1.72	0.06011	0.08471	0.10379	1.00	1.41	1.73
		55	0.06747	0.09524	0.11603	1.00	1.41	1.72	0.06708	0.09406	0.11537	1.00	1.40	1.72
10	40	25	0.0555	0.07813	0.0959	1.00	1.41	1.73	0.06076	*	0.10445	1.00	*	1.72
		35	0.05603	0.07879	0.09708	1.00	1.41	1.73	0.06576	0.09261	0.11353	1.00	1.41	1.73
		45	0.06155	0.08642	0.10616	1.00	1.40	1.72	0.07103	0.09958	0.12235	1.00	1.40	1.72
		55	0.06734	0.09524	0.11643	1.00	1.41	1.73	0.07221	0.10182	0.12287	1.00	1.41	1.70
20	20	25	0.05247	0.07498	0.09037	1.00	1.43	1.72	0.05247	0.07392	0.09037	1.00	1.41	1.72
		35	0.05445	0.0784	0.09498	1.00	1.44	1.74	0.05682	0.08037	0.098	1.00	1.41	1.72
		45	0.06063	0.08498	0.10458	1.00	1.40	1.72	0.06932	0.09708	0.11945	1.00	1.40	1.72
		55	0.06734	0.09498	0.11643	1.00	1.41	1.73	0.07182	0.10116	0.12419	1.00	1.41	1.73
30	20	25	0.04102	0.058	0.10603	1.00	1.41	2.58*	0.04125	0.05865	0.07147	1.00	1.42	1.73
		35	0.04037	0.0505	0.07129	1.00	1.25*	1.77	0.05445	0.07748	0.09458	1.00	1.42	1.74
		45	0.06195	0.08748	0.10774	1.00	1.41	1.74	0.06261	0.0884	0.10774	1.00	1.41	1.72
		55	0.07274	0.10287	0.12577	1.00	1.41	1.73	0.06484	0.09142	0.11222	1.00	1.41	1.73

*Outlier or missing peak results due to low Bragg peak intensity

The internal water concentration in the MO-AzoPS dispersions was calculated using geometric packing analysis with the lattice parameter calculated from SAXS results. The fraction of lipid (φ_l) was first calculated from:[17]

$$\varphi_l = 2A_0\left(\frac{l}{a}\right) + \frac{4}{3}\pi\chi\left(\frac{l}{a}\right)^3 \qquad \text{Eq. S6}$$

where A_0 is the dimensionless area of minimal surface per unit cell (2.345 for the primitive cubic phase),[17] l is the monolayer thickness (approximated as that of pure MO, 17.6 Å[18]), χ is the Euler characteristic (-4 for the primitive cubic phase)[17] and a is the calculated lattice parameter from the SAXS data. Subsequently, the fraction of internal water (φ_w) was calculated as 1- φ_l.

Table S8. Calculated values for the final water fraction (φ_w) in the cubosomes for the AzoPS in the *trans* and *cis*-PSS isomers with varying initial water concentration in the parent, bulk LLC phase.

**Initial water concentration (wt%)**	**Final water fraction (φ_w)**			
	**$C_6AzoC_4E_4$**		**$C_8AzoC_8E_4$**	
	trans	***cis***	***trans***	***cis***
10	0.508	0.447	0.498	0.474
20	0.500	0.448	0.498	0.455
30	0.497	0.465	0.497	0.462
40	0.494	0.459	0.458	0.430

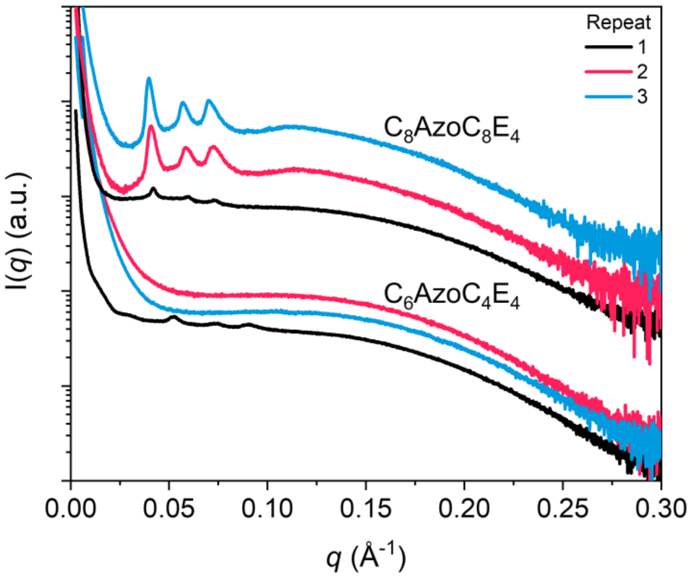

Figure S9. SAXS curves for repeat dispersions made to the same composition of MO-AzoPS (30 wt%)-initial water (20 wt%) for both $C_6AzoC_4E_4$ and $C_8AzoC_8E_4$ (T = 25 °C). Dispersions with $C_6AzoC_4E_4$ show low or no Bragg peak intensity. In contrast, dispersions with $C_8AzoC_8E_4$ show sharp Bragg peaks in the ratio √2:√4:√6 across all three repeat samples. Curves have been offset and error bars removed for clarity.

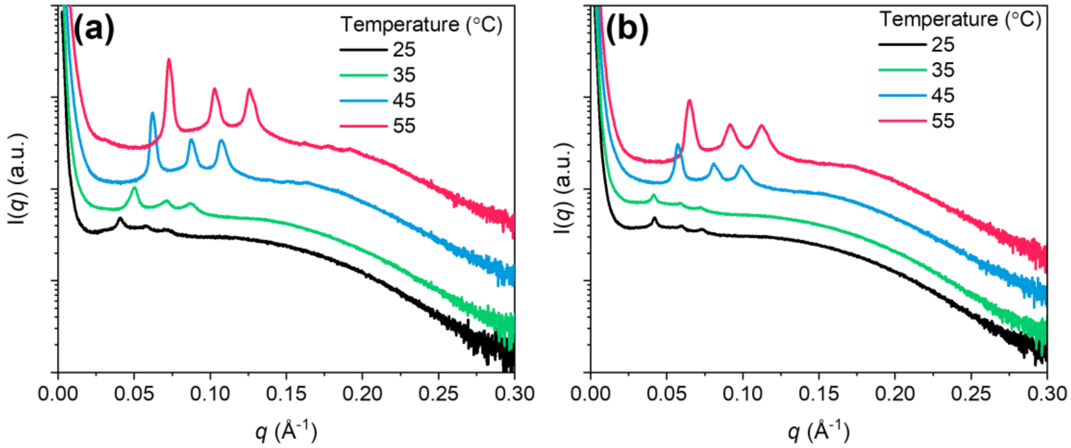

Figure S10. Effect of temperature on cubosome stability. SAXS curves for dispersions of MO-AzoPS (30 wt%)-initial water (20 wt%) for (a) $C_6AzoC_4E_4$ and (b) $C_8AzoC_8E_4$ at increasing temperatures. At each temperature, samples were equilibrated until the SAXS patterns showed no further changes.

9 UV-Vis absorption spectra

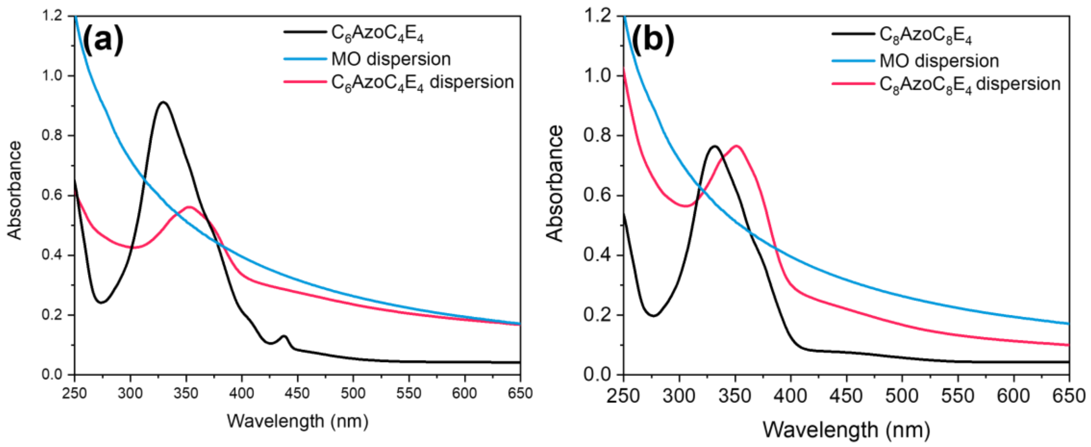

Figure S11. The UV-Vis absorption spectrum of *trans*-AzoPS becomes red-shifted upon incorporation into MO dispersions for both (a) $C_6AzoC_4E_4$ and (b) $C_8AzoC_8E_4$. Samples were diluted in water to prevent saturation of the detector to: 57 μM ($C_6AzoC_4E_4$ in water), 46 μM (MO-water (20wt%) dispersion), 69 μM (MO-$C_6AzoC_4E_4$ (20 wt%)-water (10wt%) dispersion), 68 μM ($C_8AzoC_8E_4$ in water) and 69 μM (MO-$C_8AzoC_8E_4$ (20 wt%)-water (10wt%) dispersion).

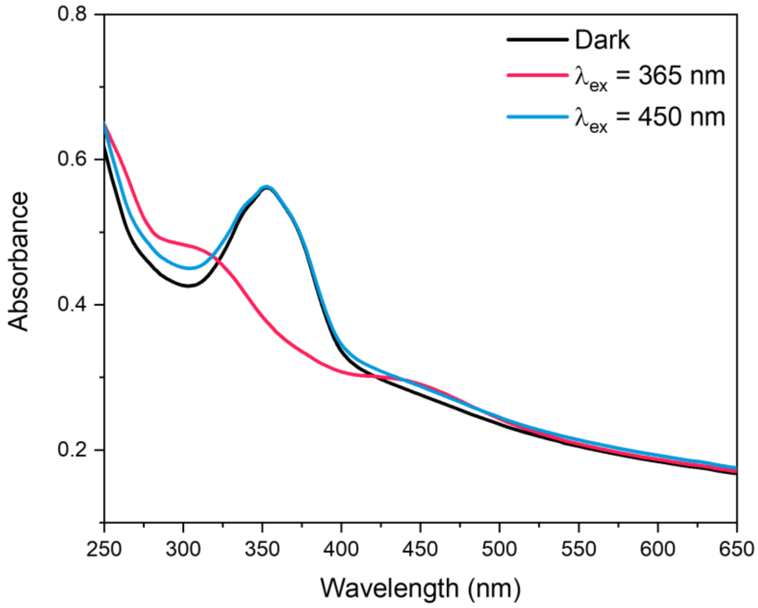

Figure S12. UV-Vis absorption spectra showing isomerization between *trans* and *cis* states for a dispersion of MO-$C_6AzoC_4E_4$ (20 wt%)-water (10 wt%), diluted to 69 μM, when: stored in the dark, after irradiation with UV light (5 minutes) and subsequent blue light irradiation (5 minutes).

10 AzoPS isomerization kinetics

Isomerization of the azobenzene moiety can be described using first-order rate kinetics, where the rate constant, k_{cis}, can be calculated from the changing the *trans* absorbance peak over time:[19]

$$k_{cis}\,t = \ln\frac{A_0 - A_{PSS}}{A_t - A_{PSS}} \qquad \text{Eq. S5}$$

where A_0, A_{PSS} and A_t are the absorbance at the *trans*-λ_{max} before irradiation, in the PSS and after time *t* respectively. The same analysis can be used for reverse isomerization to calculate k_{trans}.

UV-Vis absorption spectra for dispersions of MO containing 10 wt% water and 20 wt% AzoPS were collected after sequential irradiation with UV (Figure S13a and S14a) and blue light (Figure S13b and S14b). Plots with linear regions fitted through the origin were used to calculate k_{cis} and k_{trans} for the forward and reverse isomerization processes, respectively. The half-life for photoconversion, τ, was then calculated from the inverse of *k* (Table S9).

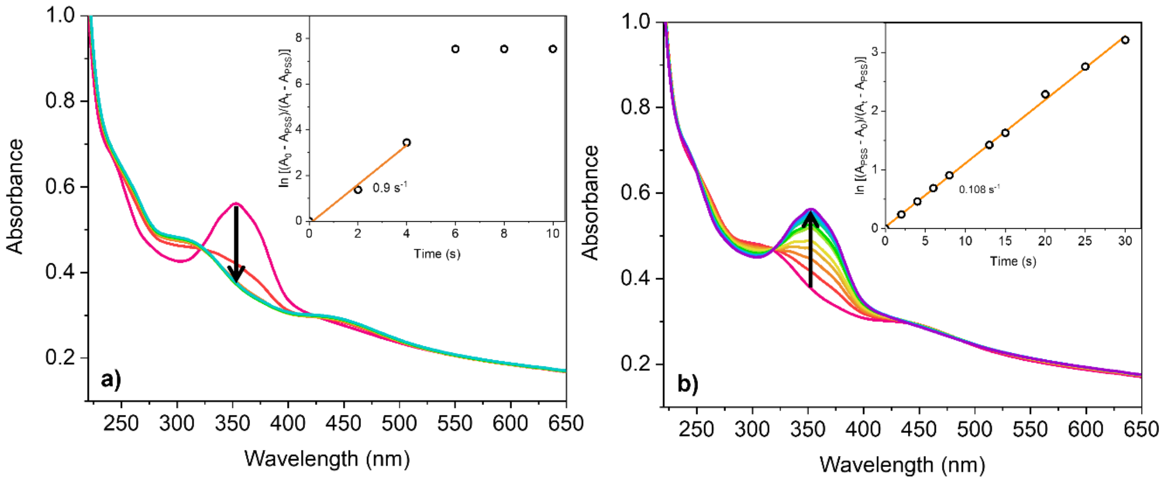

Figure S13. UV-Vis absorption spectra and first-order rate kinetics plots for dispersions of MO containing 20 wt% $C_6AzoC_4E_4$ and 10 wt% water, diluted to 69 μM, as a function of time under (a) UV irradiation (λ = 365 nm) and (b) reversal under blue irradiation (λ = 450 nm), after 5 minutes of initial UV irradiation. First-order kinetic plots were used to determine the rate constants k_{cis} and k_{trans} as described in the text. Note that fast conversion under UV irradiation results in a low number of data points and low accuracy of results using this experimental set-up, however, it can still be concluded that switching occurs within seconds.

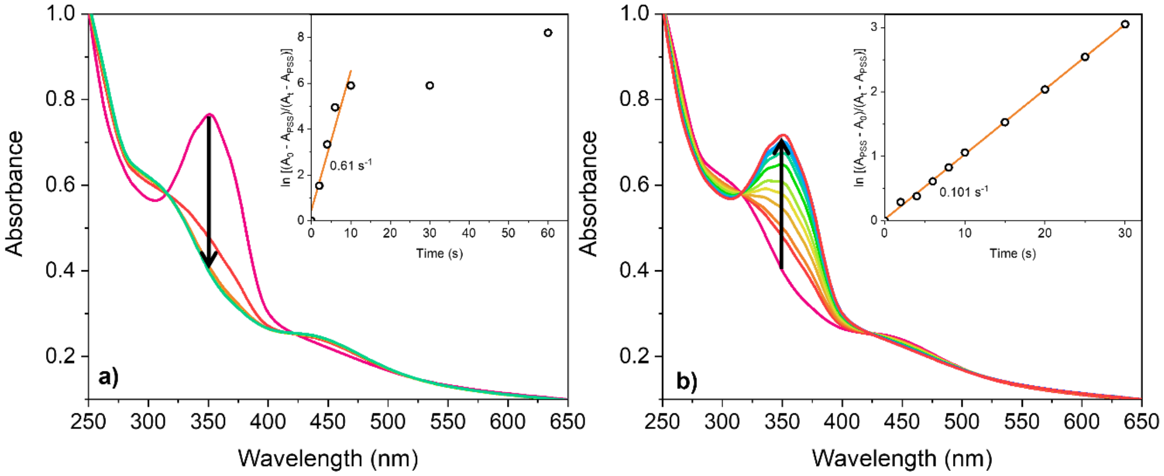

Figure S14. UV-Vis absorption spectra and first-order kinetic plots for dispersions of MO containing 20 wt% $C_8AzoC_8E_4$ and 10 wt% water, diluted to 69 μM, as a function of time under (a) UV irradiation (λ = 365 nm) and (b) blue irradiation (λ = 450 nm) reversal under blue irradiation (λ = 450 nm), after 5 minutes of initial UV irradiation. First-order kinetics plots were used to determine the rate constants k_{cis} and k_{trans} as described in the text. Note that fast conversion under UV irradiation results in a low number of data points and low accuracy of results using this experimental set-up, however, it can still be concluded that switching occurs within seconds.

Table S9. Timescales for isomerization within MO-AzoPS dispersions. First-order rate constants, *k*, and conversion half-lives, τ, for photoisomerization of MO-AzoPS (20 wt%)-water (10 wt%) LLC dispersions on irradiation with UV, *cis* (λ_{ex}=365 nm) and blue, *trans* (λ_{ex}=455 nm) light and thermal *cis* to *trans* lifetime on storage in the dark ($\tau_{thermal}$).

Sample	k_{cis} (s^{-1})	τ_{cis} (s)	k_{trans} (s^{-1})	τ_{trans} (s)	$\tau_{thermal}$ (hr)
MO-$C_6AzoC_4E_4$ dispersion	0.86 ± 0.10	1.2 ± 0.1	0.108 ± 0.001	9.23 ± 0.12	7.5 ± 0.6
MO-$C_8AzoC_8E_4$ dispersion	0.61 ± 0.09	1.6 ± 0.3	0.101 ± 0.001	9.91 ± 0.10	2.0 ± 0.1

On irradiation, a photostationary state (PSS) containing a mixture of both *trans* and *cis* isomers that dynamically switch between the two states is formed.[20] The degree of isomerization within the PSS on UV irradiation can be calculated as:[20]

$$ID_{cis} = \frac{A_0(365) - A_{PSS}(365)}{A_0\,(365)} \times 100\% \qquad \text{Eq. S6}$$

where A_0(365) is the absorbance at λ = 365 nm before irradiation and A_{PSS}(365) is the absorbance at the same wavelength in the *cis*-PSS.

Similarly, the degree of isomerisation for reverse isomerization can be calculated:

$$ID_{trans} = \frac{A_{PSS}\ (365)}{A_0\ (365)} \times 100\% \qquad \text{Eq. S7}$$

where $A_{PSS}(365)$ now refers to the absorbance of the *trans*-PSS at 365 nm. The results for the isomerization degrees obtained are summarized in Table S10. It is worth noting that the high background, $\propto \lambda^{-4}$, due to Rayleigh scattering from the particles results in lower calculated values for ID_{cis} than expected.

Table S10. Degree of isomerization in the photostationary state for MO-AzoPS (20 wt%)-water (10 wt%) LLC dispersions on irradiation with UV, ID_{cis} (λ_{ex}=365 nm) and blue, ID_{trans} (λ_{ex}=455 nm) light.

Sample	ID_{cis} (%)	ID_{trans} (%)
MO-$C_6AzoC_4E_4$ dispersion	34	100
MO-$C_8AzoC_8E_4$ dispersion	50	94

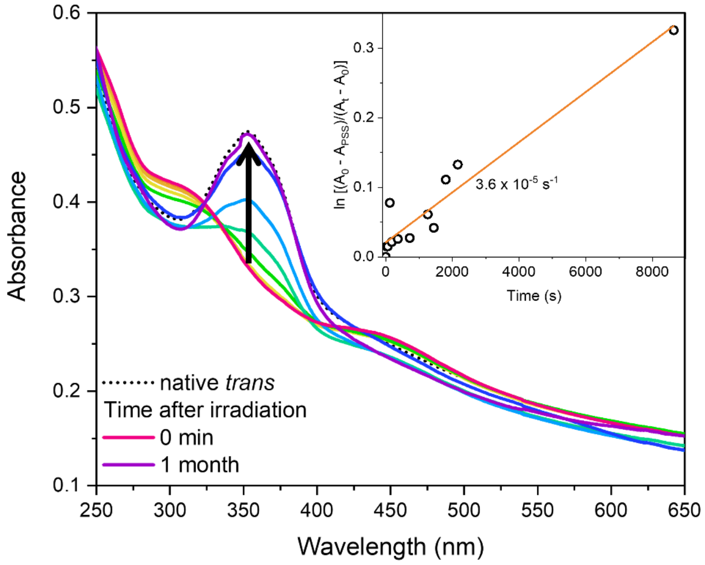

Figure S15. Thermal reversion of *cis*-$C_6AzoC_4E_4$ in a MO dispersion. UV-Vis absorption spectra of a MO-$C_6AzoC_4E_4$ (20 wt%)-water (10 wt%) LLC dispersion in the native, *trans* state, after irradiation with UV light (365 nm) and storage in the dark after subsequent time intervals. Insert shows the first-order rate kinetics plot used to determine the rate constant for the process and thermal half-life on storage.

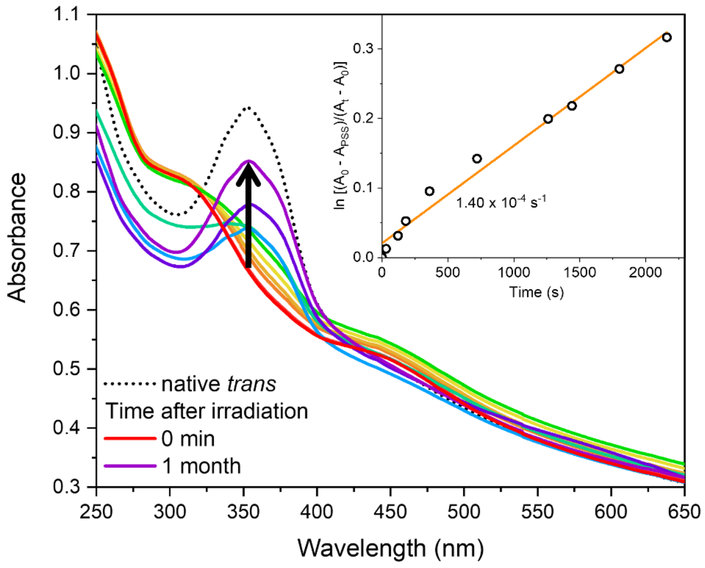

Figure S16. Thermal reversion of *cis* $C_8AzoC_8E_4$ in a MO dispersion. UV-Vis absorption spectra of a MO-$C_8AzoC_8E_4$ (20 wt%)-water (10 wt%) LLC dispersion in the native, *trans* state, after irradiation with UV light (365 nm, 5 minutes) and storage in the dark after subsequent time intervals. Insert shows the first-order rate kinetics plot used to determine the rate constant for the process and thermal half-life on storage.

11 SAXS data for isomerized dispersions

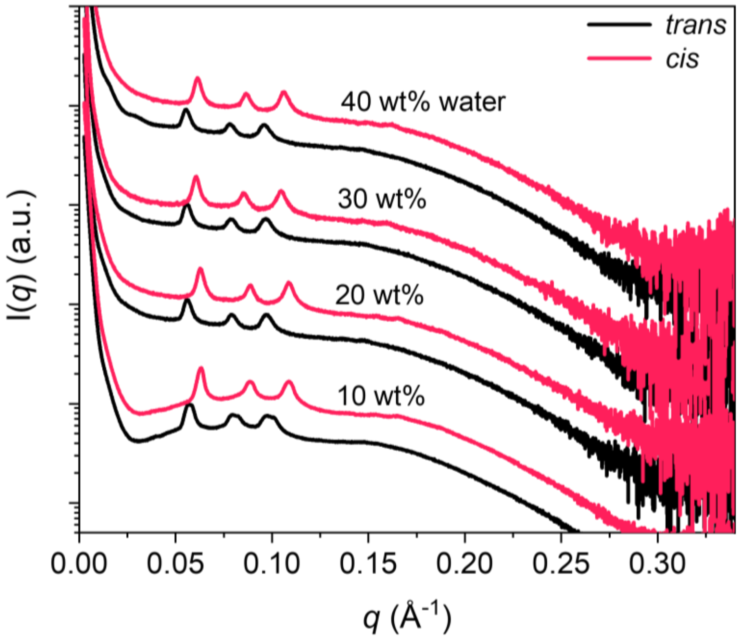

Figure S17. Change in SAXS curves on isomerization within MO-$C_6AzoC_4E_4$ (10 wt%)-water dispersions with increasing concentration of initial water. *q* peak positions are in the ratio of √2:√4:√6, indicating the presence of the inverse bicontinuous primitive cubic (*Im3m*) phase. Curves have been offset and error bars removed for clarity.

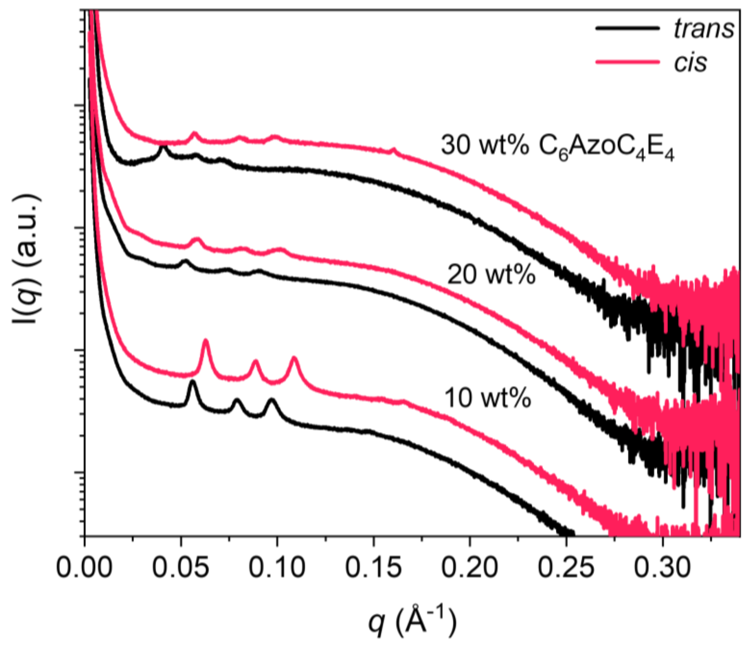

Figure S18. Change in SAXS curves on isomerization within MO-$C_6AzoC_4E_4$ -water (20 wt%) dispersions with increasing concentration of $C_6AzoC_4E_4$. q peak positions are in the ratio of √2:√4:√6, indicating the presence of the inverse bicontinuous primitive cubic (*Im3m*) phase. Curves have been offset and error bars removed for clarity.

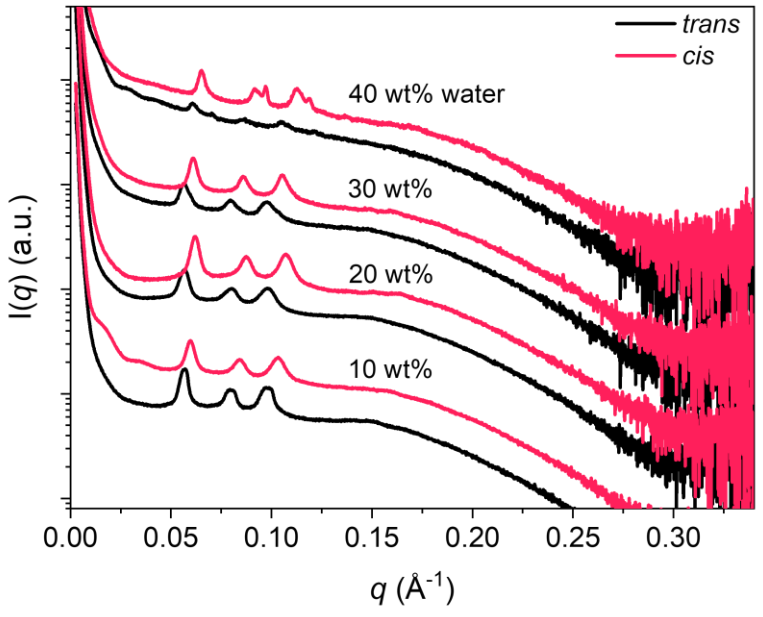

Figure S19. Change in SAXS curves on isomerization within MO-$C_8AzoC_8E_4$ (10 wt%)-water dispersions with increasing concentration of initial water. q peak positions are in the ratio of √2:√4:√6, indicating the presence of the inverse bicontinuous primitive cubic (*Im3m*) phase. For the sample with 40 wt% water, the secondary and tertiary peaks are split into 2, with a ratio √4:√6. Curves have been offset and error bars removed for clarity.

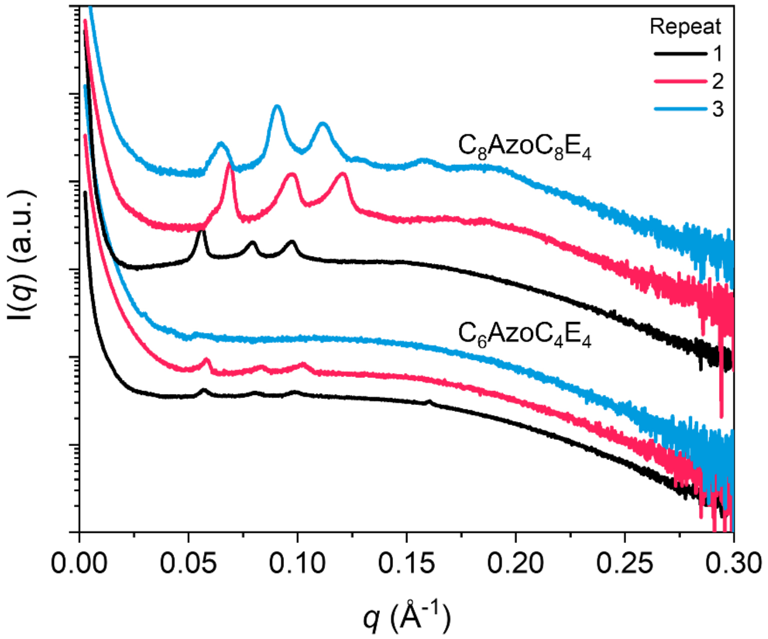

Figure S20. SAXS curves for repeat dispersions made to the same composition of MO-AzoPS (30 wt%)-initial water (20 wt%) for both $C_6AzoC_4E_4$ and $C_8AzoC_8E_4$ in the isomerised, *cis* state. Dispersions with $C_6AzoC_4E_4$ show low or no Bragg peak intensity. In contrast, dispersions with $C_8AzoC_8E_4$ show sharp Bragg peaks in the ratio √2:√4:√6 across all 3 repeats. Curves have been offset and error bars removed for clarity.

12 Dynamic light scattering for isomerized dispersions

Table S11. Size and polydispersity in isomerized dispersions. Variation in hydrodynamic diameter, D_H, and polydispersity index, *PDI*, for dispersions of MO-water (20 wt%) and varying AzoPS concentration. Note only one measurement was taken for each sample to reduce the effects of reverse isomerization from the light beam.

AzoPS (wt%)	D_H (nm)		PDI	
	$C_6AzoC_4E_4$	$C_8AzoC_8E_4$	$C_6AzoC_4E_4$	$C_8AzoC_8E_4$
10	234	257	0.30	0.39
20	234	211	0.43	0.17
30	218	571	0.30	0.31

13 References

1. Houston, J. E.; Kelly, E. A.; Kruteva, M.; Chrissopoulou, K.; Cowieson, N.; Evans, R. C. Multimodal Control of Liquid Crystalline Mesophases from Surfactants with Photoswitchable Tails. *Journal of Materials Chemistry C* **2019**, *7* (35), 10945–10952.

2. Kelly, E. A.; Houston, J. E.; Evans, R. C. Probing the Dynamic Self-Assembly Behaviour of Photoswitchable Wormlike Micelles in Real-Time. *Soft Matter* **2019**, *15* (6), 1253–1259.

3. Cowieson, N. P.; Edwards-Gayle, C. J. C.; Inoue, K.; Khunti, N. S.; Doutch, J.; Williams, E.; Daniels, S.; Preece, G.; Krumpa, N. A.; Sutter, J. P.; Tully, A. D.; Terrill, N. J.; Rambo, R. P. Beamline B21: High-Throughput Small-Angle X-Ray Scattering at Diamond Light Source. *Journal of Synchrotron Radiation* **2020**, *27*, 1438–1446.

4. Rambo, R. P. *ScÅtter*. http://www.bioisis.net/tutorial/9.

5. International Organisation for Standardisation (ISO). *Particle Size Analysis - Dynamic Light Scattering* (ISO Standard No. 22412:2017), 2017.

6. Briggs, J.; Chung, H.; Caffrey, M. The temperature-composition phase diagram and mesophase structure characterization of the monoolein/water system. *J. Phys. II*, **1996**, 6 (5), 723-751.

7. Israelachvili J. N.; Mitchell D. J.; Ninham, B. W. Theory of self-assembly of hydrocarbon amphiphiles into micelles and bilayers. *J. Chem. Soc., Faraday Trans. 2* **1976**, 72, 1525-1568

8. Durchschlag, H.; Zipper, P. Calculation of the partial volume of organic compounds and polymers. In: *Ultracentrifugation*; Lechner, M.D., Eds.; Progess in Colloid & Polymer Science, vol 94, **1994**; pp 20-39

9. Tanford, C. Micelle shape and size. *J. Phys. Chem.* **1972**, 76, 3020-3024

10. Kulkarni, C. V. Calculating the 'Chain Splay' of Amphiphilic Molecules: Towards Quantifying the Molecular Shapes. *Chem. Phys. Lipids* **2019,** 218, 16-21

11. Takeshita, K.; Hirota, N.; Terazima, M. Enthalpy changes and reaction volumes of photoisomerization reactions in solution: azobenzene and p-coumaric acid. *J. Photochem. Photobiol. A: Chem.* **2000**, 134, 103-109

12. Merino, E.; Ribagorda, M. Control over molecular motion using *cis-trans* photoisomerization of the azo group. *Beilstein J. Org. Chem.* **2012**, 8, 1071-1090

13. Agapito, F.; Cabral, B. J. C.; Simões, J. A. M. Carbon-hydrogen bond dissociation enthalpies in ethers: a theoretical study. *J. Mol. Struct. THEOCHEM* **2005,** 719, 109-114

14. Rosen, M. J.; Cohen, A. W.; Dahanayake, M.; Hua, X.Y. Surface and thermodynamic properties of 2-dodecyloxypoly(ethenoxyethanol)s, C12H25(OC2H4)xOH, in aqueous solution. *J. Phys. Chem*. **1982**, 86, 541-545

15. Guinier, A.; Fournet, G. Small angle scattering of X-rays. *John Wiley & Sons, Inc.*, **1955**

16. Schnablegger, H. & Singh, Y. The SAXS guide: getting acquainted with the principles. *Anton Paar,* **2017**

17. Leung, S. S. W.; Leal, C. The stabilization of primitive bicontinuous cubic phases with tunable swelling over a wide composition range. *Soft Matter* **2019**, 15, 1269-1277

18. Chung, H., Caffrey, M. The curvature elastic-energy function of the lipid-water cubic mesophase. *Nature* **1994**, 368, 224-226

19. Sierocki, P.; Maas, H.; Dragut, P.; Richardt, G.; Vogtle, F.; De Cola, L.; Brouwer, F.; Zink, J. I. Photoisomerization of azobenzene derivative in nanostructured silica. *J. Phys. Chem. B* **2006**, 110, 24390–24398

20. Peng, S.; Guo, Q.; Hartley, P. G.; Hughes, T. C. Azobenzene Moiety Variation Directing Self-Assembly and Photoresponsive Behavior of Azo-Surfactants. *J. Mat. Chem. C* **2014**, *2* (39), 8303–8312
